# N-helix and Cysteines Inter-regulate Human Mitochondrial VDAC-2 Function and Biochemistry*[Fn FN2]

**DOI:** 10.1074/jbc.M115.693978

**Published:** 2015-10-20

**Authors:** Svetlana Rajkumar Maurya, Radhakrishnan Mahalakshmi

**Affiliations:** From the Department of Biological Sciences, Molecular Biophysics Laboratory, Indian Institute of Science Education and Research, Bhopal 462023, India

**Keywords:** gating, ion channel, membrane protein, protein folding, thermodynamics, voltage-dependent anion channel (VDAC), N-terminal domain

## Abstract

Human voltage-dependent anion channel-2 (hVDAC-2) functions primarily as the crucial anti-apoptotic protein in the outer mitochondrial membrane, and additionally as a gated bidirectional metabolite transporter. The N-terminal helix (NTH), involved in voltage sensing, bears an additional 11-residue extension (NTE) only in hVDAC-2. In this study, we assign a unique role for the NTE as influencing the chaperone-independent refolding kinetics and overall thermodynamic stability of hVDAC-2. Our electrophysiology data shows that the N-helix is crucial for channel activity, whereas NTE sensitizes this isoform to voltage gating. Additionally, hVDAC-2 possesses the highest cysteine content, possibly for regulating reactive oxygen species content. We identify interdependent contributions of the N-helix and cysteines to channel function, and the measured stability in micellar environments with differing physicochemical properties. The evolutionary demand for the NTE in the presence of cysteines clearly emerges from our biochemical and functional studies, providing insight into factors that functionally demarcate hVDAC-2 from the other VDACs.

## Introduction

The voltage-dependent anion channels (VDACs)^3^ are the most abundant outer mitochondrial membrane β-barrel proteins in eukaryotes. In humans, they exist as three isoforms (hVDAC-1, -2, and -3) and together govern the passage of metabolites and ions across the outer mitochondrial membrane (1, 2). hVDAC-1 is 10- and 100-fold more abundant than hVDAC-2 and -3, respectively, and is the most characterized VDAC protein (1, 3). Crystal and NMR structures for hVDAC-1 are concurrent with a 19-stranded β-barrel, bearing an N-terminal helix (NTH) lining the pore interior (4–6). hVDAC-2 and hVDAC-3 share 72 and 69% sequence identity to hVDAC-1, and are expected to adopt similar structural scaffolds (1).

hVDAC-1 is known to exhibit the highest ion channel activity, which is voltage-gated by the NTH (5, 7). Furthermore, hVDAC-1 and -3 possess pro-apoptotic functions (1), whereas hVDAC-2 is gaining popularity for its anti-apoptotic property (inhibition of BAK function) (8). The recent crystal structure of zebrafish VDAC-2 revealed that the NTH lines the pore interior in a manner resembling hVDAC-1 (9). A definitive placement of this amphipathic helix in all VDAC isoforms has been the subject of constant debate. There is experimental evidence for the NTH lining the pore (4–6, 9–11), located outside the pore and toward the cytosol (12, 13) forming a part of the barrel wall (14, 15) and the remaining membrane adsorbed (16). It was seen that the NTH required a specialized hydrophobic or amphipathic environment to attain its structure in aqueous conditions (17–19). Taken together, the data reflect the highly dynamic nature of the NTH.

The hVDAC-1 NTH is essential during apoptosis and cytochrome *c* release, as it interacts with the anti-apoptotic factors Bcl-2 and hexokinase-1 (20). Conversely, NTH is dispensable for metabolite exchange, cell growth, mitochondrial targeting of the protein, as well as barrel oligomerization (17, 20). NTH deletion results in complete loss of VDAC voltage dependence (7, 20–24), suggesting that the NTH is a voltage-gating sensor. It is, however, unclear whether the NTH-less channel remains in the high conductance (20, 23) or the subconductance state (7, 21, 22, 24). Models that explain the NTH-mediated gating and hVDAC-1 subconductance state at >±30 mV include: (i) helix translocation into the barrel, causing pore closure (25); (ii) alteration of NTH-pore interaction due to change in NTH structure (26, 27); (iii) translocation of the helix from the pore wall to the center (5, 6); (iv) ellipsoidal barrel formation upon NTH removal (7); and (v) no structural rearrangement during gating (28).

**FIGURE 1.**
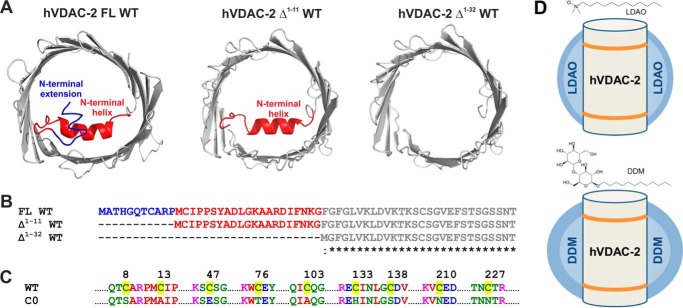
**hVDAC-2 N-terminal deletion mutants and detergent systems used in this study.** *A,* the I-TASSER (57) modeled structure of the hVDAC-2 barrel (*gray*) is shown on the *left*, with NTE in *blue* and NTH in *red. Middle* and *right panels* show the I-TASSER modeled structure of hVDAC-2 Δ^1–11^ and Δ^1–32^ WT, respectively. *B*, multiple sequence alignment of the N-terminal mutants of hVDAC-2, and color coded according to *A. C*, comparison of hVDAC-2 WT and C0 sequences, highlighting the nine cysteines of WT in *yellow*, and showing the corresponding replacements in C0. All residues have been color coded according to their physicochemical properties. *D*, schematic representation of the hVDAC-2 barrel in LDAO (*upper panel*) and DDM (*lower panel*) micelles. The hydrophobic core of the micelles is shown in *light blue* and the polar headgroups in *dark blue*. The hydrophobic core of the protein is demarcated by *orange lines*. Note the spherical and oblate shapes of LDAO and DDM micelles, respectively. Chemical structures of LDAO and DDM are also provided to highlight the size of the headgroups.

It is evident that the NTH of VDAC-1 can possess diverse functional properties and structural characteristics. In hVDAC-2, the NTH carries an additional 11-residue sequence that we term “N-terminal extension” (NTE, residues 1–11) (Fig. 1*A*). NTE is absent in the other VDAC isoforms, and is implicated in hVDAC-2 channel activity, as well as resistance to reactive oxygen species (ROS) (29). The role of the NTH and NTE of hVDAC-2 were recently examined in the context of channel function and gating (24, 29, 30). However, our understanding of the N-helix (NTH + NTE) function in hVDAC-2 is remarkably poor when compared with hVDAC-1. Furthermore, the possibility that the helix plays additional structural roles in hVDAC-2 has not yet been examined.

In this study, we address the role of hVDAC-2 NTH and NTE in the function and stability of this transmembrane β-barrel. Furthermore, we evaluate the significance of our findings in micellar systems and lipid bicelles. We demonstrate that the removal of NTE and NTH disrupts barrel voltage sensing and stability in a manner that is considerably influenced by the presence of the hVDAC-2 cysteine residues. Additionally, our data supports a biophysical role for the N-helix in barrel refolding kinetics and points to an evolutionary significance for why hVDAC-2 requires an extended N-terminal segment.

## Experimental Procedures

### 

#### 

##### Production and Refolding of hVDAC-2 WT (Wild Type) and Its Mutants

Human *vdac*-2 gene in pET-3b (31) was used as the template for the generation of *c*0-pET-3b, through site-directed mutagenesis. hVDAC-2 constructs lacking NTE (Δ^1–11^) and NTE + NTH (Δ^1−32^) were cloned from these parent constructs. Cys-less full-length (FL C0) had the following mutations: C8S, C13A, C47E, C76T, C103A, C133H, C138S, C210N, and C227N (see Fig. 1*C*). *Escherichia coli* BL21(DE3) cells were transformed with these plasmids, and the proteins were expressed as inclusion bodies. We followed the previously established protocol (31) for protein purification.

Refolding of hVDAC-2 WT and its mutants was achieved by rapid 10-fold dilution of 250 μm denatured protein (in 6 m GdnHCl and 10 mm DTT) in 65 mm LDAO (lauryldimethylamine oxide) or 19.5 mm DDM (dodecyl β-d-maltoside) prepared in Buffer A (100 mm NaCl, 10 mm DTT, 50 mm phosphate buffer, pH 7.2) at 4 °C. Refolding reactions were incubated at 4 °C for 5 h, and aggregated protein, if any, was removed by centrifugation at 15,500 × *g* for 1 h at 4 °C. The final refolded protein 5× stocks thus prepared contained 25 μm protein in 65 mm LDAO or 19.5 mm DDM in Buffer A; residual GdnHCl (0.6 m) was not removed.

Bicelles of varying *q* (*q* = [long chain lipid; DMPC]/[short chain lipid; LDAO or DDM]) ranging from 0.00015 to 1, were generated by subjecting a DMPC (1,2-dimyristoyl-*sn*-glycero-3-phosphocholine) gradient (0.01 to 20 mm), prepared in 65 mm LDAO or 19.5 mm DDM in Buffer A, to five freeze-thaw cycles (31). 250 μm denatured protein (in 6 m GdnHCl and 10 mm DTT) was refolded by 10-fold dilution in the bicelles preparations, as described for the micelles. Samples were additionally dialyzed against Buffer A to remove residual GdnHCl and centrifuged to remove aggregated protein, to generate the 5× refolded stock.

Unless specified otherwise, all stock proteins were diluted 5-fold in Buffer A (without DTT), for the various measurements.

##### Channel Conductance Measurements

Refolded protein (5× refolded protein stock (25 μm) in 65 mm LDAO in Buffer A supplemented with 0.1% cholesterol and 1% Triton X-100 (32)) was added to the *cis* side of a planar lipid bilayer made up of 12.5 mg/ml of DiPhPC (diphytanoyl phosphatidylcholine) + 0.1% cholesterol painted across a 200-μm aperture. The *cis* and *trans* chambers contained 10 mm HEPES buffer, pH 7.4, 5 mm CaCl_2_, and 1 m KCl (32). All bilayer recordings and data analyses were carried out using reported methods (33). Briefly, the channel insertion events were recorded at a holding voltage of +10 mV with a filtering frequency of 400 Hz and voltage dependence was studied using a 3 mHz triangular voltage ramp between ±60 mV at a filtering frequency of 15 kHz. 20–100 active channels were used for voltage ramp measurements.

The conductance (*G*/*G*_max_) and open probability (*P*_open_) plots were calculated for the channel reopening part of the ramp (33). The steepness of the voltage dependence (*n*) and the voltage at which half the channels are open (V_0_) were obtained using Equation 1 (33).

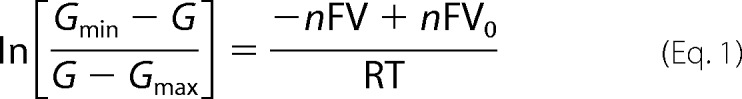
 Here, *G* is the conductance at any voltage V, *G*_min_ and *G*_max_ are the minimum and maximum conductance seen at higher and lower voltages, respectively, *F* is the Faraday constant, *R* is the gas constant in joules, and *T* is the temperature in kelvin.

##### Peptide Synthesis and Sample Preparation

The NTH sequence of hVDAC-2 WT (residues 12–32; see Fig. 1*B*), was synthesized by Fmoc (*N*-(9-fluorenyl)methoxycarbonyl) chemistry, using established protocols (34), with a Cys-13 → Ala replacement. Two sample types with different protein:peptide ratios (1:10–1:1000) were prepared: (i) peptide was added to the unfolded protein and co-refolding was done; (ii) peptide was separately folded in LDAO by mixing, and added to the refolded protein stock, or directly in the *cis* and *trans* chamber, whereas carrying out the planar lipid bilayer experiments.

##### Barrel Refolding Kinetics Measurements

Refolding kinetics was monitored using Trp fluorescence anisotropy (*r*) by rapid 10-fold dilution of 250 μm denatured protein (in 6 m GdnHCl and 10 mm DTT) into 65 mm LDAO or 19.5 mm DDM at 4 °C (31). A λ_ex_ = 295 nm and λ_em_ = 340 nm were used to acquire data every ∼15.74 s. The experiment dead time was ∼20–25 s (31). Data were fitted to a single exponential function to derive the refolding rate (*k*_f_) (31).

##### Equilibrium Unfolding and Refolding

Intrinsic Trp fluorescence was used to measure equilibrium refolding in GdnHCl (35). 25 μm protein in 65 mm LDAO or 19.5 mm DDM in Buffer A (refolded stock) or Buffer A supplemented with 6 m GdnHCl (for LDAO) or 4 m GdnHCl (for DDM) (unfolded stock) were diluted 5-fold in a GdnHCl gradient. The final reactions contained 5 μm protein, 13 mm LDAO, or 3.9 mm DDM in Buffer A containing 2 mm DTT. The progress of all the reactions were monitored for 16 h in LDAO and 72 h in DDM using λ_ex_ = 295 nm and λ_em_ = 310–400 nm, at 25 °C. In LDAO, prolonged incubation (more than 1 h) resulted in protein precipitation in GdnHCl concentrations of ∼1–3 m. The fluorescence intensity at 340 nm (λ_em-max_ of folded protein) was used to calculate the unfolded fraction (*f*_U_) and fitted to a two-state equation to obtain the equilibrium free energy of unfolding (Δ*G*_U_^0^) at 0 m denaturant, *m* value, and *C_m_* (35). In conditions wherein hysteresis was observed, apparent free energy of unfolding ((Δ*G*_app_^0^) and apparent unfolding cooperativity (*m*_app_) were derived (36).

##### Anisotropy, Quenching, and Lifetime Measurements

Trp fluorescence anisotropy (*r*) was acquired at λ_em_ = 340 nm using λ_ex_ = 295 nm, slit width of 5 nm, and integration time of 5 s over three trials to achieve a standard error <2% (31). Acrylamide quenching measurements of Trp fluorescence were recorded at 25 °C using established protocols (31), to derive the Stern-Volmer constant (*K*_SV_). Trp lifetimes were measured using time-correlated single photon counting using λ_ex_ = 292 nm and the data were collected at 340 nm. Fits of the data to a triple exponential function provided amplitude fractions (α*_i_*) and corresponding lifetimes (τ*_i_*). The average lifetime (<τ>) was derived using Equation 2.

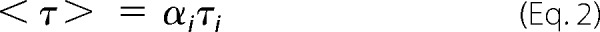
 Bimolecular quenching constant (*k_q_*) and apparent rotational correlation time (τ*_c_*) were calculated using Equations 3 and 4.

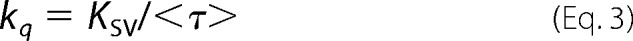


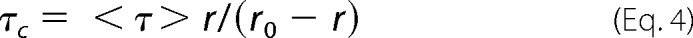
 Here *r_0_* was taken as 0.3 for indole moiety.

##### Thermal Denaturation and Protein Stability

Far-UV circular dichroism (CD) wavelength scans were obtained using a scan rate of 100 nm/min, 0.1 cm path length, 1 nm bandwidth, and 1 s response time, at 4 °C. Raw CD values (θ) in degrees were converted to mean residue ellipticity (MRE; θ_MRE_) using Equation 5.

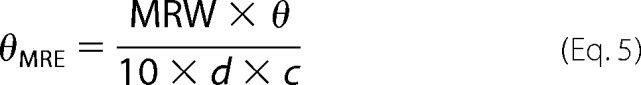
 Here, MRW = *M*(*N*-1), *M* is molecular weight of the protein in Da and *N* is the number of residues in the protein, *d* is the path length in cm, and *c* is the protein concentration in g/ml.

Thermal denaturation was monitored at 215 nm (MRE_215_) at a ramp rate of 1 °C/min, with sampling carried out at every 1 °C from 4 to 95 °C (31). Data were converted to *f*_U_ (31) and fitted with a two-state equation (37) to derive *T_m_* (midpoint of thermal denaturation) and unfolding cooperativity. For thermal denaturation in DMPC:LDAO and DMPC:DDM systems, the refolded protein stocks were diluted 5-fold in Buffer A (without DTT) to achieve a DMPC gradient of 2–4000 μm, in 13 mm LDAO or 3.9 mm DDM containing 5 μm protein. These samples were subjected to thermal denaturation, and *f*_U_ was calculated (31). The *T_m_*_-start_ (temperature at which thermal denaturation is initiated) was derived using reported methods (35).

##### Differential Scanning Calorimetry Measurements

Phase transition temperatures of DMPC in vesicular and bicellar systems were monitored using Microcal VP-differential scanning calorimetry microcalorimeter. DMPC bicelles (2–4000 μm) in 13 mm LDAO or 3.9 mm DDM were made without DTT as described in the previous section. The samples were subjected to thermal denaturation from 4 to 60 °C, at a scan rate of 60 °C/h and acquisition was carried out in high gain mode using 1 s filtering. The data were blank corrected, normalized, and processed using Microcal Origin software.

##### Data Analysis and Validation

All activity measurements were averaged over at least 4 independent recordings and spectroscopic measurements at least twice, using freshly refolded samples. Unless otherwise specified, graphs represent average data, and the error bars have been omitted for clarity. Parameters (*T_m_*, *T*_*m*-start_, unfolding cooperativity, *n*, and *V*_0_) have been derived from fits to independent datasets, with the error bars representing the standard deviation between independent experiments. The thermodynamic parameters (*C_m_*, *m* value, and (Δ*G*_U_^0^) were derived from fits to averaged data with the error bars representing the goodness of fit.

## Results

### 

#### 

##### hVDAC-2 Δ^1−32^ Shows Loss of Voltage Dependence in Lipid Bilayers and Δ^1–11^ Barrel Is Voltage Sensitized

The role of NTH in voltage sensing of hVDAC-1 is well established (7, 20, 22, 24). However, the requirement of the additional 11-residue NTE in hVDAC-2 is unclear. In the I-TASSER-predicted model of hVDAC-2 (Fig. 1*A*), NTE exists as a random coil, and this region may form minimal interactions with the channel wall. NTE may play a role in protection from ROS (29), but its role in regulating channel conductance, voltage gating, or acting as a docking site for the Bcl-2 family of proteins is unclear. We therefore probed the functional and physiological properties of hVDAC-2 WT FL and the N-helix truncations in black lipid membranes, by designing hVDAC-2 constructs Δ^1–11^ and Δ^1–32^ (Fig. 1, *A* and *B*). Additionally, hVDAC-2 is known to have unusually high cysteine content. Cysteines are rare in proteins, and their unusual abundance in hVDAC-2 suggests that they may be functionally important. Hence, we assessed the interplay of cysteines and NTH(+NTE), by generating C0 constructs (Fig. 1*C*).

**FIGURE 2.**
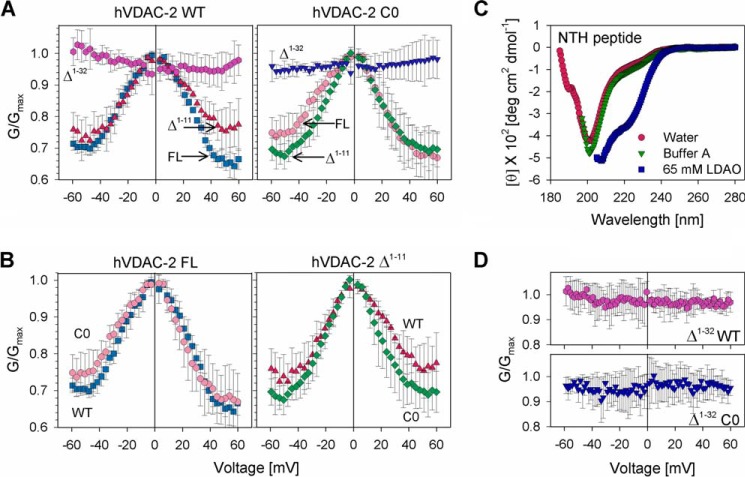
**Channel conductance measurements of hVDAC-2 and its mutants in lipid bilayers.** *A*, *G*/*G*_max_ plots for WT proteins (*left panels*) and C0 constructs (*right panels*), obtained in response to a voltage gradient ranging from +60 to −60 mV in DiPhPC + 0.1% cholesterol membrane. The data shows the importance of the NTH in voltage gating of hVDAC-2. In both the WT and C0 barrels loss of voltage dependence is seen upon deletion of the N-helix. WT constructs: FL (*square*), Δ^1–11^ (*triangle*), and Δ^1–32^ (*hexagon*); C0 constructs: FL (*circle*), Δ^1–11^ (*diamond*), and Δ^1–32^ (*inverted triangle*). *B,* comparison of the *G*/*G*_max_ plots of WT and C0 mutants of FL hVDAC-2 (*left panel*) and Δ^1–11^ hVDAC-2 (*right panel*). *C,* the NTH peptide is unstructured (random coil conformation) in both water and Buffer A, and shows a helical conformation only in 65 mm LDAO (in Buffer A), when measured using far-UV CD. The data suggests that NTH can interact with the lipid environment in hVDAC-2, which can promote NTH structuring. *D,* exogenously supplemented NTH peptide is unable to restore voltage-dependent gating in Δ^1–32^ mutants. Experiments were carried out with protein:peptide ratios ranging from 1:10 to 1:1000 and results for 1:1000 ratio is shown. *Error bars* in panels *A*, *B,* and *D* are derived from at least four independent experiments.

We used multichannel membranes to monitor the response of hVDAC-2 to triangular voltage ramps (33). In VDAC-1, separate gating mechanisms at positive and negative voltages gives rise to asymmetric voltage dependence in DiPhPC bilayers (38). *In vivo* function for this asymmetry is unknown. Bilayer properties, strand deletions, disulfides, and NTH mutations affect this asymmetry, through directed insertions, conformational changes, or gating charge neutralization (11, 23, 39, 40). In hVDAC-2 FL, a quantitative comparison of the normalized conductance (Fig. 2, *A* and *B*) reveals asymmetric gating, whereas the deletion of NTE (Δ^1–11^) elicits a more symmetrical response to the applied voltage (Fig. 2*B*). NTE deletion changes the voltage dependence in the positive arm for WT and negative arm for C0 (Fig. 2*B*). Hence, depending on the presence of cysteines, NTE deletion shows opposite effects on hVDAC-2, with an overall similar outcome of symmetrized voltage sensing. Based on the *n*FV_0_ values (discussed below), the removal of NTE perturbs hVDAC-2 asymmetric gating through a conformational change in the barrel.

Complete removal of NTE + NTH (Δ^1–32^) abolishes the voltage dependence of hVDAC-2 (Fig. 2*A*), similar to previous observations for other VDACs (7, 20–24). Supplementing a synthetic NTH separately to the Δ^1–32^ barrel does not revive channel gating characteristics for both WT and C0 (Fig. 2*D*), although the peptide adopts a helical conformation (Fig. 2*C*). Our data reveals that a helical structure of the N-helix is insufficient for its proper function and voltage gating, its proper placement within the barrel in *cis* is mandatory.

**FIGURE 3.**
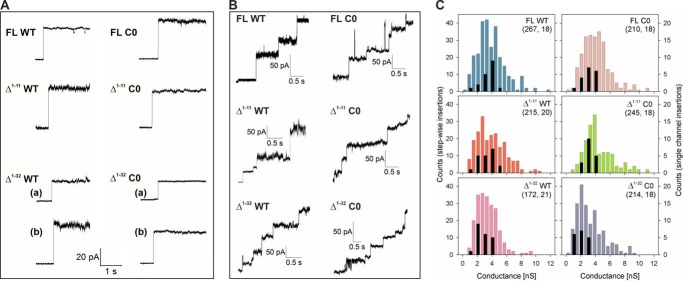
**Channel insertion events of hVDAC-2 and its mutants.** *A,* representative single channel insertion events of hVDAC-2 mutants in DiPhPC + 0.1% cholesterol membrane at 10 mV. Two events are shown for Δ^1–32^ mutants, representing insertion at ∼2 nS ((*a*), subconductance state) and ∼4 nS ((*b*), open state). *B,* representative stepwise insertion events of hVDAC-2 mutants. *C,* histograms obtained from channel insertion events; those depicted in color are obtained from stepwise insertion events shown in *B* with the counts given on the *left*. Histograms in *black* are derived from single channel insertion events shown in *A*, with the counts shown on the *right*. Insertion of a few VDAC channels in the DiPhPC membrane promotes further channel insertion events. Hence, simultaneous insertion of 2–4 channels gives us a broad range in the conductance histogram derived from stepwise insertions. Both methods show that the removal of NTE + NTH gives rise to higher instances of channel insertions in the subconductance state. *Numbers in brackets* indicate total number of insertion events considered for deriving the histograms from stepwise and single channel insertion events, respectively.

Our single channel conductance measurements indicate that hVDAC-2 WT and C0 insert in DiPhPC membranes in mainly two different conductance states of ∼2 nS (subconductance) and ∼4 nS (open) (Fig. 3 and Table 1). The ratio of these states remains similar across FL and Δ^1–11^ constructs. Surprisingly, N-helix deleted Δ^1–32^ WT channel also shows the existence of open and subconductance states, unlike hVDAC-1, where only the subconductance state is reported (7, 21, 22, 24). The Cys-less Δ^1–32^ hVDAC-2 preferably exists in this subconductance state (Fig. 3*C* and Table 1). Our results show that the open channel state is accessible to helix-less hVDAC-2 when cysteines are present. Loss of the N-terminal helix in Δ^1–32^ WT may not always give rise to the ellipsoidal barrel with a narrow pore suggested for hVDAC-1 (7), and may be accessible predominantly to Δ^1–32^ C0.

**TABLE 1:** **Summary of electrophysiological parameters of hVDAC-2 mutants measured in DiPhPC membranes** Errors in all cases are derived from averaging independent experiments.

hVDAC-2 mutants*^a^*	Positive voltages			Negative voltages			Single channel conductance (nS)	
	*n*	V_0_*^b^*	*n*FV_0_*^c^*	*n*	V_0_*^b^*	*n*FV_0_*^c^*	Single insertions*^d^*	Stepwise insertions*^d,e^*
**FL WT (5)**	3.12 ± 0.50	28.63 ± 1.92	8.62	3.07 ± 0.47	−25.00 ± 2.64	7.40	2.43 ± 0.58 (6), 3.98 ± 0.50 (12)	2.30 ± 0.72 (5), 4.45 ± 0.57 (8)
**Δ^1–11^ WT (4)**	2.26 ± 0.39	24.15 ± 0.91	5.27	2.98 ± 0.27	−23.49 ± 0.89	6.75	2.13 ± 0.52 (8), 3.91 ± 0.46 (11)	2.50 ± 0.35 (5), 4.39 ± 0.62 (8)
**Δ^1–32^ WT**							2.20 ± 0.42 (16), 3.86 ± 0.60 (9)	2.02 ± 0.60 (11), 4.02 ± 0.35 (6)
**FL C0 (6)**	2.40 ± 0.66	21.07 ± 1.72	4.88	2.31 ± 0.68	−25.57 ± 3.68	5.70	2.30 ± 0.54 (8), 3.75 ± 0.48 (10)	2.18 ± 0.67 (4), 3.96 ± 0.57 (8)
**Δ^1–11^ C0 (6)**	2.79 ± 0.17	21.71 ± 1.51	5.84	2.69 ± 0.26	−25.45 ± 2.02	6.60	2.52 ± 0.41 (6), 3.62 ± 0.50 (12)	2.45 ± 0.80 (4), 3.77 ± 0.46 (8)
**Δ^1–32^ C0**							1.84 ± 0.43 (16), 3.28 ± 0.17 (4)	2.01 ± 0.62 (10), 4.27 ± 0.47 (6)

*^a^* Numbers in parentheses indicate the number of independent experiments conducted for the voltage ramp studies to derive *n* and V_0_.

*^b^ V*_0_ is given in mV.

*^c^ n*FV_0_ is given in kJ mol^−1^.

*^d^* The two values represent insertions segregated as “open” (∼4 nS) and “subconductance” (∼2 nS) states. Numbers in parentheses indicate the number of channels considered, and are obtained from at least three independent experiments.

*^e^* Conductance measured for the first 4–5 single channel insertions.

We calculated *n*FV_0_, which indicates the energy difference between the open and closed channel states (33). This value decreases marginally when NTE is deleted (Table 1), particularly in the WT protein. Such changes might arise from structural rearrangements in the barrel (33). We have previously reported that the association affinity of FL WT and C0 to their refolding environment depends on barrel rigidity (31). It is likely that NTE removal alters the barrel scaffold. Taken together with cysteines, we observe that NTE influences the voltage sensing of hVDAC-2, can function only in *cis*, and plays an important role in the measured barrel characteristics.

##### NTE + NTH Lowers Barrel Refolding Rates and NTE Minimizes Off-pathway Aggregates in Cys-rich WT

Our electrophysiology measurements establish that the N-helix of hVDAC-2 is crucial for voltage gating and sensing. Generally, residues conferring functionality to proteins also affect other biophysical properties such as (un)folding and stability (41). To address this, we compared the folding/unfolding characteristics of the Δ^1–11^ and Δ^1–32^ constructs to the full-length barrels. We chose two 12-C micellar environments for our folding experiments: LDAO (zwitterionic detergent with small headgroup) and DDM (non-ionic detergent with a large headgroup). LDAO forms small spherical micelles (∼17 kDa, aggregation number = 76), whereas DDM micelles are oblate (42), bulky (50–71 kDa), with a higher aggregation number (98-140). Variations in the micelle shape, size, and headgroup can create differences in the hydrophobic radius (42) (see Fig. 1*D*), ability to incorporate cylindrical or elliptical barrels, and display different protein-micelle interactions. LDAO is structurally analogous to a chemical chaperone (32), and preserves the VDAC structure seen in bilayers (43). DDM is a known stabilizing agent of membrane proteins (44) and maintains the native (functional) protein state.

**FIGURE 4.**
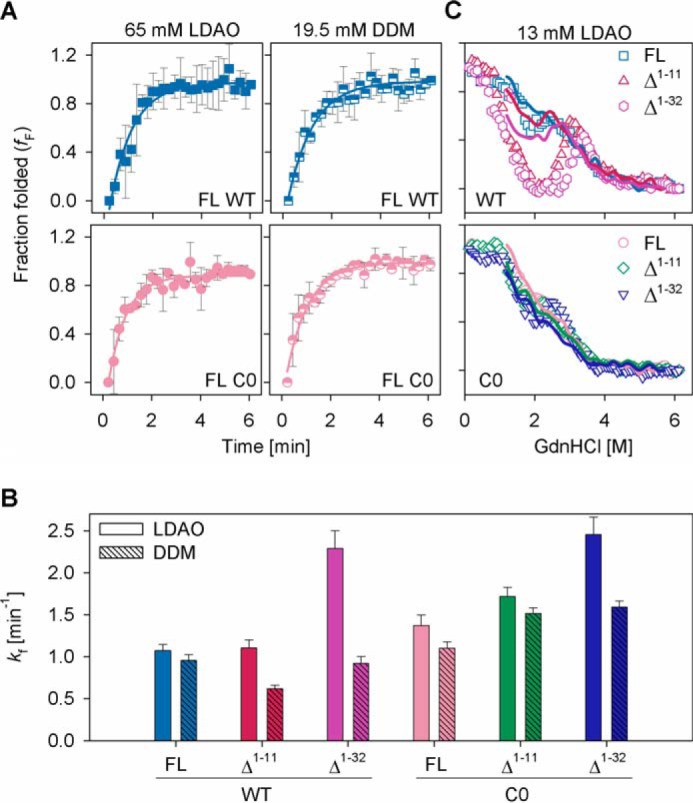
**Re/unfolding experiments in LDAO and DDM micelles.** *A,* refolding kinetics of hVDAC-2 WT (*upper panel*) and C0 (*lower panel*) in 65 mm LDAO (*left*) and 19.5 mm DDM (*right*) measured using changes in Trp fluorescence anisotropy. *Solid lines* represent fits to a single exponential function, used to derive the rate of refolding (*k_f_*) shown in *B*. The differences obtained upon comparison of FL and Δ^1–11^ with Δ^1–32^, and WT with C0 constructs in LDAO or DDM are significant (*p* value: <0.05, derived using one-way analysis of variance method). However, taking into consideration the rapid refolding kinetics of all hVDAC-2 constructs and the experimental dead time (see “Experimental Procedures”), these data must be interpreted with extreme caution. *C*, chemical denaturation studies in 13 mm LDAO using GdnHCl, at 25 °C. The unfolding and refolding curves are represented using *symbols* and *lines*, respectively, and were recorded after 16 h of incubation at 25 °C. *Open symbols* are used and error bars are hidden for clarity. The symbol/color schemes are retained from Fig. 2*A*. The Δ^1–11^ and Δ^1–32^ WT mutants are most affected, and considerable protein aggregation is observed. Although C0 and its mutants also aggregate, the fluorescence intensity is not drastically affected, possibly due to the different nature of aggregates in this case. *Error bars* in *A* denote S.D. values derived from independent experiments and in *B* show the goodness of fit.

We used 65 mm LDAO or 19.5 mm DDM for our experiments. These concentrations were chosen to achieve high refolding efficiency, similar micelle number, and comparable secondary structure content for all constructs. The refolding efficiency does not increase further with an increase in the detergent concentration (data not shown). We probed the refolding rates for all the constructs by measuring the intrinsic fluorescence anisotropy (31) of the four interface tryptophans (Fig. 4*A*). The refolding rates are very fast, and we can capture them only through changes in anisotropy. We see an overall increase in the refolding rates upon deletion of NTH + NTE. In the presence of LDAO, a marginal increase is seen in rates for some constructs, when compared with DDM (Fig. 4*B*). Our data indicates that in the absence of the N-helix, barrel refolding is kinetically rapid and favorable. NTE + NTH and cysteines together impede refolding. Global comparison of refolding rates of all WT constructs against those for C0 constructs in both LDAO and DDM shows an increase of >20% in the folding rates of the C0 constructs. Hence, our WT constructs display slower folding kinetics than their C0 counterparts (Fig. 4*B*).

The elevated folding rates of hVDAC-2 Δ^1–32^ mutants, particularly in LDAO for WT, suggest that the N-helix introduces frustration in the folding pathway for β-barrel formation. NTE + NTH may prevent the formation of native contacts in the barrel or stabilize a refolding intermediate. Hence, why were residues 1–11 retained in the protein? To understand this, we carried out equilibrium refolding measurements of all barrels in LDAO. Interestingly, considerable non-disulfide aggregates form at intermediate GdnHCl concentrations in all proteins (Fig. 4*C*). Furthermore, Δ^1–11^ WT and Δ^1–32^ WT are the only barrel forms to be substantially affected by off-pathway protein aggregation. Such aggregates have been observed for both soluble and membrane proteins (45), and in hVDAC-2, occur at denaturant concentrations that allow the formation of folding intermediate.

Our study shows that in hVDAC-2 WT, NTE(+NTH) lowers the barrel refolding rate to minimize off-pathway protein aggregation. The concomitant absence of aggregates in this system (discussed later), shed valuable light on hVDAC-2 folding mechanism. Cysteines have high hydropathy values (46), and exposed thiols in the refolding intermediate can augment protein association and result in nonspecific disulfides. Therefore, NTE(+NTH) of Cys-rich hVDAC-2 can play a crucial role in directing proper barrel formation, by interacting with exposed hydrophobic surfaces and establishing transient disulfides. Our assumption is supported by the altered aggregate characteristics of the refolding intermediate observed between 1 and 3 m GdnHCl in hVDAC-2 C0 (Fig. 4*C*). We conclude that NTE is important for the proper refolding of hVDAC-2 WT and plays a crucial role in solubilizing the refolding intermediate.

##### NTE and NTH Impart Thermodynamic Stability to hVDAC-2 in DDM with Structural Rearrangements Evident in LDAO

We next measured the contribution of NTE, NTH, and cysteines to hVDAC-2 thermodynamic behavior, using equilibrium denaturation measurements. We obtain irreversible aggregation of hVDAC-2 upon prolonged incubation in LDAO (Fig. 4*C*). Furthermore, the refolding process prior to the onset of aggregation is three-state, with a prominent refolding intermediate (Δ^1–32^ > Δ^1–11^ > FL for occurrence of the intermediate). However, barrel unfolding can be adequately explained using a two-state model. The resultant hysteresis in the system allows us to estimate only the Δ*G*_app_^0^ in LDAO (Table 2, discussed later).

**TABLE 2:** **Apparent thermodynamic parameters in 13 mm LDAO** Samples were incubated for 1 h at 25 °C before data acquisition. Errors in all cases represent goodness of fit.

Mutants	Δ*G*_app_^0^	*m*_app_	*C_m_*
	*kcal mol*^−*1*^	*kcal mol*^−*1*^ *m*^−*1*^	*m*
**FL WT**	4.38 ± 0.14	−1.39 ± 0.04	3.15 ± 0.02
**Δ^1–11^ WT**	5.76 ± 0.19	−1.62 ± 0.05	3.55 ± 0.01
**Δ^1–32^ WT**	6.35 ± 0.24	−1.79 ± 0.06	3.54 ± 0.01
**FL C0**	3.21 ± 0.11	−1.13 ± 0.04	2.68 ± 0.02
**Δ^1–11^ C0**	2.57 ± 0.08	−1.02 ± 0.03	2.50 ± 0.02
**Δ^1–32^ C0**	4.65 ± 0.14	−1.55 ± 0.05	3.00 ± 0.01

**FIGURE 5.**
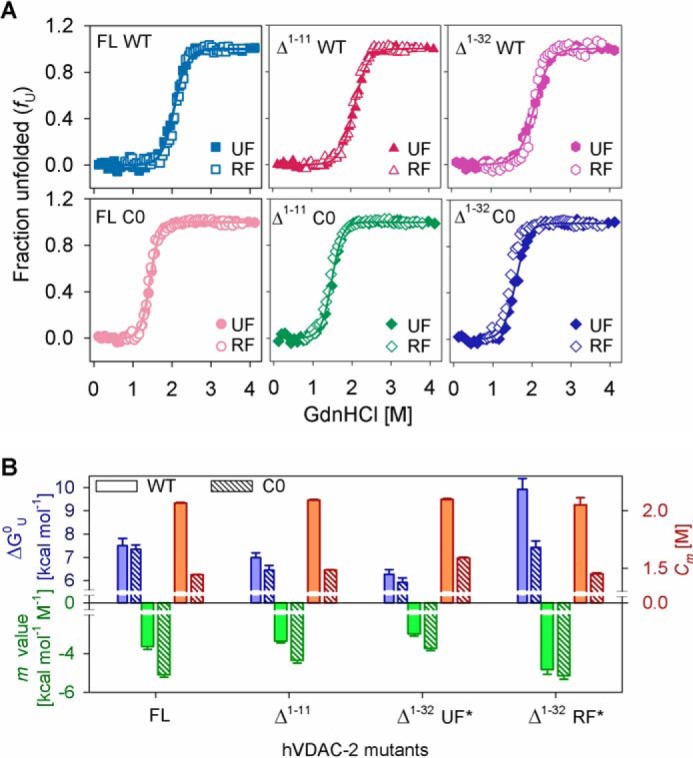
**Chemical denaturation studies of hVDAC-2 in DDM and LDAO.** *A,* the unfolding and refolding curves of hVDAC-2 mutants after a 24-h incubation at 25 °C in DDM shows no hysteresis in FL and Δ^1–11^ constructs. Mild hysteresis is observed in Δ^1–32^ constructs, and apparent unfolding (UF*) and refolding (RF*) thermodynamic parameters were derived. *Solid lines* represent fits to the two-state equation (used to derive the thermodynamic parameters in *B*) and have been only shown for the unfolding curves. *B*, summary of thermodynamic parameters in 3.9 mm DDM. The free energy of unfolding is shown in *blue bars*, *m* value in *green*, and *C_m_* in *red. Asterisk* (*) in Δ^1–32^ mutants represents the apparent thermodynamic parameters. For convenient comparison, the sign of Δ*G*_app_^0^ and *m* values for RF* for Δ^1–32^ are reversed. In *panel A* errors have not been shown for purpose of clarity and those in *panel B* represent goodness of fit.

The unfolding and refolding profiles for all proteins in DDM show cooperative two-state transitions from the micelle-solvated refolded barrel to the GdnHCl-solvated unfolded state (Fig. 5*A*). Protein aggregation is considerably diminished and is observed only upon prolonged incubation (>48 h) (data not shown). An appreciable amount of hysteresis is obtained only in the Δ^1–32^ constructs. This stems from differences in the cooperativity of the refolding *versus* unfolding processes (low unfolding *m* value; Fig. 5*B*). In DDM, NTE deletion causes barrel destabilization by ∼0.5 kcal/mol (for WT) and ∼0.9 kcal/mol (for C0), whereas obliterating the N-helix further lowers barrel stability by ∼1.3 kcal/mol for both proteins (FL *versus* unfolding Δ*G*_app_^0^ of Δ^1–32^) (Fig. 5*B*). We have previously observed that in LDAO, FL C0 relies on intra-protein interactions to maintain structural integrity (31). Lowered *C_m_* values in FL C0 (Fig. 5*B*) supports a similar behavior of the Cys-less barrel in DDM. Truncation of the N-helix increases the *C_m_*, whereas lowering the unfolding cooperativity, suggesting that intra-protein interactions are replaced with protein-micelle interactions in these proteins. Our data, therefore, underlie the importance of NTE and NTH for thermodynamic stability of hVDAC-2, and connects the functional role to the biophysical property of the protein.

As we observe hysteresis and protein precipitation during hVDAC-2 refolding in LDAO, we extracted information on barrel-micelle interaction strengths from only the unfolding measurements. Although unfolding is a two-state event, the *m* values in Table 2 are unusually low for an ∼32 kDa protein. Such low *m* values indicate that the unfolding titration corresponds to a hidden three-state process. Therefore, the Gibbs free energy is underestimated in our experiments (47). In line with observations in DDM, the *C_m_* of WT constructs are higher than the C0 counterparts (Table 2). The observed increase in Δ*G*_app_^0^ when the N-helix is deleted arises from an increase in both *m*_app_ and *C_m_*. In this case, Δ*G*_app_^0^ embodies the solvability of the refolded protein, which, in turn, reflects the protein-micelle interaction strengths. As seen for DDM, removal of the N-helix therefore increases the affinity of the barrel for its lipid milieu.

When compared with DDM, the decreased cooperativity of hVDAC-2 constructs in LDAO shows that the denaturant gains slower access to the barrel in this micelle, thus increasing the transition region of unfolding. To examine if this is due to protein-LDAO interactions, we compared the accessibility and degree of solvent exposure of the four intrinsic tryptophans. The *K*_SV_ (represents fluorophore-quencher interaction kinetics), *k_q_* (represents quenching efficiency or degree of tryptophan exposure), *r* (measures the ability of fluorophore to re-orient in the excited state), and τ*_c_* (measures the rate of fluorophore rotational diffusion) are provided in Table 3.

**TABLE 3:** **Tryptophan fluorescence properties of hVDAC-2 and its mutants**

Micelle	Mutants	hVDAC-2 WT					hVDAC-2 C0				
		<τ>*^a^*	*K*_sv_*^b^*	*k_q_**^c^*	*r**^d^*	τ*_c_**^e^*	<τ>*^a^*	*K*_sv_*^b^*	*k_q_**^c^*	*r**^d^*	τ*_c_**^e^*
**13 mm LDAO**	**FL**	2.50	4.56	1.83	0.124	1.76	3.08	5.67	1.84	0.130	2.37
	**Δ^1–11^**	2.58	5.09	1.97	0.137	2.16	3.10	5.44	1.75	0.128	2.34
	**Δ^1–32^**	2.52	5.28	2.09	0.146	2.39	3.00	5.33	1.78	0.133	2.39
**3.9 mm DDM**	**FL**	2.50	3.79	1.52	0.162	2.94	2.99	4.24	1.42	0.153	3.12
	**Δ^1–11^**	2.42	3.62	1.50	0.162	2.85	3.00	4.28	1.43	0.153	3.13
	**Δ^1–32^**	2.35	3.64	1.55	0.168	3.00	3.04	4.14	1.36	0.163	3.63

*^a^* <τ> given in 10^9^ s.

*^b^ K*_sv_ in m^−1^.

*^c^ k_q_* in 10^9^ m^−1^ s^−1^.

*^d^ r* in arbitrary units.

*^e^* τ*_c_* in 10^9^ s.

*K*_SV_ values are lower in DDM compared with LDAO, for both proteins. Similar lifetimes in both micellar systems, however, suggest that the solvent-exposed indoles in LDAO maintain a rigid local environment, whereas the flexible indoles in DDM micelles are occluded from the solvent. This is supported by the higher τ*_c_* in DDM, suggesting that the bulky headgroup of DDM occludes Trp from fluorescence quenchers. Hence, LDAO can form better interactions with hVDAC-2, and facilitates optimal positioning of the indole at the interface. Comparison of the secondary structure content of the refolded proteins using far-UV CD wavelength scans further supports that hVDAC-2 adopts slightly higher β-sheet content in LDAO (compare the MRE_215_ values in LDAO and DDM in Table 4).

**TABLE 4:** **Thermal denaturation parameters of hVDAC-2 in 13 mm LDAO and 3.9 mm DDM** Errors in all cases are obtained from averaging independent experiments.

Parameters	Mutants	hVDAC-2 WT		hVDAC-2 C0	
		LDAO	DDM	LDAO	DDM
MRE_215_ × 10^3^ (deg cm^2^ dmol^−1^)	FL	−12.7 ± 0.12	−11.6 ± 0.41	−13.1 ± 0.36	−11.1 ± 0.59
	Δ^1–11^	−12.7 ± 1.91	−11.4 ± 0.60	−13.7 ± 0.20	−10.8 ± 0.75
	Δ^1–32^	−11.7 ± 2.21	−11.4 ± 0.24	−11.6 ± 0.08	−11.1 ± 0.91
*T_m_* (°C)	FL	69.34 ± 0.35	64.33 ± 0.92	71.20 ± 0.31	62.09 ± 1.71
	Δ^1–11^	65.57 ± 1.36	64.76 ± 2.02	68.37 ± 0.39	61.31 ± 1.12
	Δ^1–32^	63.80 ± 1.21	69.59 ± 2.82	69.40 ± 0.36	69.06 ± 0.89
Cooperativity (kcal mol^−1^)	FL	65.56 ± 2.22	59.13 ± 11.66	90.97 ± 8.62	59.99 ± 12.16
	Δ^1–11^	44.06 ± 4.43	35.84 ± 05.81	99.24 ± 3.11	58.28 ± 02.46
	Δ^1–32^	34.12 ± 1.79	28.10 ± 12.45	61.19 ± 6.16	43.15 ± 11.64
*T_m_*_-start_ (°C)	FL	60.85 ± 0.78	53.00 ± 1.70	65.15 ± 1.71	51.20 ± 0.85
	Δ^1–11^	54.02 ± 1.39	50.00 ± 0.86	60.90 ± 0.85	52.52 ± 2.64
	Δ^1–32^	47.59 ± 0.83	49.99 ± 2.56	59.83 ± 0.24	56.26 ± 1.85

By and large, in LDAO, Trp accessibility in WT constructs increase when NTE and NTH are deleted, whereas Trp accessibility in C0 counterparts decrease. Within the same micelle system, N-helix and cysteines have complementary effects, promoting barrel-micelle interactions in WT. Furthermore, in LDAO and DDM, lowered *K*_SV_ and τ*_c_* for the WT constructs indicates lipid-solvated indoles, whereas C0 indoles are more solvent accessible. Such local changes in the Trp environment of WT can arise from structural rearrangements driven by changes in the shear number. This has been speculated for VDACs (48). Therefore, a lowered protein-micelle interaction in C0 explains our observed thermodynamic and spectroscopic properties.

##### Thermal Denaturation Measurements Reveal the Role of NTE and NTH in Stabilizing a Compact Barrel

**FIGURE 6.**
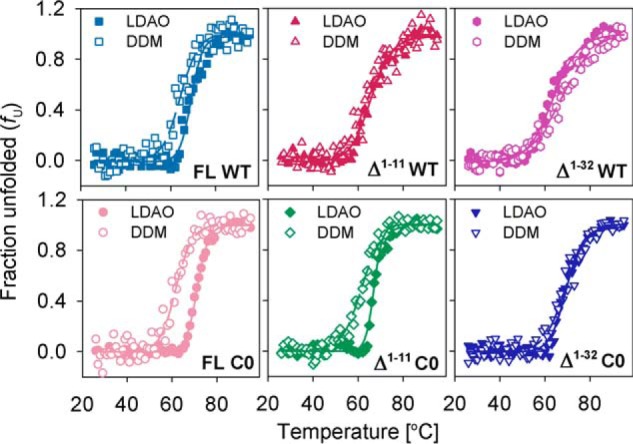
**Thermal denaturation studies of hVDAC-2.** Data were obtained from 13 mm LDAO (*filled symbols*) and 3.9 mm DDM (*open symbols*) and the parameters derived from the data and fits are summarized in Table 4. *Dashed* and *solid lines* denote fits to the two-state equation for LDAO and DDM, respectively. Overall, removal of the NTH drastically affects the cooperativity in both LDAO and DDM. Error bars have been omitted for clarity.

Our previous studies on thermal denaturation of hVDAC-2 revealed that the native barrel is susceptible to heat-mediated irreversible unfolding, which is tightly associated with aggregation (49). A measure of the changes in the secondary structure through far-UV CD (Fig. 6 and Table 4) provides information on scaffold stability and protein-micelle interactions. Assessment of the observed reduction at 215 nm (Fig. 6) suggests a micelle- and N-helix dependence to the aggregation event. The *T_m_* is comparable for all constructs (Table 4). Overall, the values are marginally higher in LDAO than DDM, as expected. Examination of the thermal denaturation-derived parameters for the NTE + NTH deletions reveals that the significant contribution of the N-helix is in stabilizing the barrel scaffold. The *T_m_*_-start_ (35) is lower in most of the constructs lacking NTE or NTH (Table 4). Furthermore, removal of NTE lowers the unfolding cooperativity of hVDAC-2 WT in both LDAO and DDM micelles by ∼20 kcal/mol (Table 4). Complete deletion of the N-helix further affects the unfolding cooperativity by ∼10 kcal/mol. hVDAC-2 Δ^1–32^ C0 barrel also exhibits lower unfolding cooperativity by ∼15 kcal/mol in DDM and ∼30 kcal/mol in LDAO micelles, although the deletion of NTE barely changes the cooperativity in C0 (compare FL C0 and Δ^1–11^ C0 in Table 4).

This change in cooperativity represents the change in enthalpy during protein unfolding. Refolded hVDAC-2 enthalpy lies in the range of 0.13–0.33 kcal/mol/residue, which is similar to other membrane proteins, but is significantly less than values observed for soluble proteins (50). Low enthalpy for membrane proteins are presumed to stem from a structured denatured state, contributions solely from the extra-membrane segments and/or a buried hydrophobic region upon unfolding (50, 51). In hVDAC-2, barrel unfolding leads to aggregation that buries a considerable region of the protein. These aggregates are not structured (data not shown). Hence, our enthalpy measurements reflect only the change associated with solvent-exposed regions, and mainly involve the N-helix.

Among the FL constructs, C0 exhibits higher enthalpy than WT (Table 4) that suggests a higher exposed surface area in this protein. This observation is in agreement with our fluorescence measurements that C0 shows poor protein-micelle interactions. When the solvent-accessible N-helix is deleted, the enthalpy is lowered. The N-helix of hVDAC-2 is structured (Fig. 2*C*) and establishes favorable energetic contacts (30), contributing to the high enthalpy of FL constructs. NTE deletion drastically reduces the enthalpy for only the WT barrel (Table 4), convincing us that this segment exists in a solvent-exposed structured state. On the contrary, C0 barrel rigidity and the lack of cysteines can cause the NTE to remain unstructured, as a result of which we see almost no change in enthalpy upon its deletion (Table 4). The NTH is amphipathic, can dock onto the barrel wall (4–6, 9, 10), and lower the solvent accessibility of these side chains. Hence, the deletion of NTH shows only small enthalpy changes in both WT and C0 (Table 4).

Across our hVDAC-2 deletions, a part of the decrease in enthalpy can also stem from removal of the contacts that the N-helix forms with the transmembrane β-barrel. Another likely explanation is the destabilization of the refolded state of Δ^1–11^ and Δ^1–32^ constructs; hence, fewer stabilizing interactions are formed. The latter explanation is also supported by our *n*FV_0_ values and chemical denaturation studies. A clear picture that emerges when we consider the unfolding cooperativity, enthalpy, *T_m_*, and *T_m_*_-start_, is that NTE and NTH are critical factors for the formation of a well compacted hVDAC-2. Additionally, from the chemical and thermal denaturation data, we find that NTE adopts a soluble structured conformation in WT hVDAC-2, whereas in C0, it can remain unstructured.

##### Refolding of hVDAC-2 in High Bicelle q Is Dependent on N-helix, with Micelles and Bicelles Showing Comparable Characteristics

The behavior of membrane proteins in micelles is believed to be different from lipid bilayers. However, in VDACs, studies have affirmed that the barrel structure in LDAO and DMPC are similar (43). Nonetheless, whereas micelles are dynamic entities, lipid bilayers exert transverse and lateral forces, due to hydrophobic mismatch and packing pressure at the membrane-protein junction. To assess the requirement of NTE and NTH for hVDAC-2 *in membrana*, we used membrane bicelles of various *q*.

**FIGURE 7.**
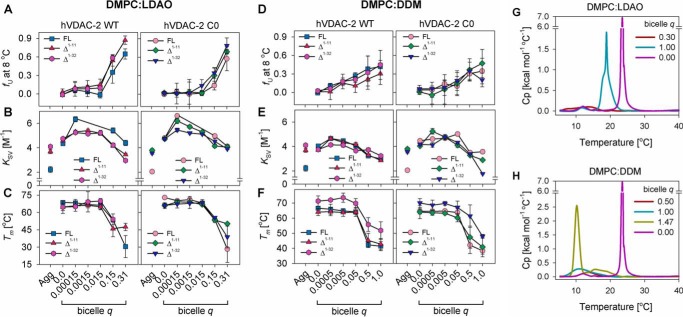
**Stability of hVDAC-2 in DMPC:LDAO and DMPC:DDM bicelles.** *A* and *D,* comparison of the unfolded fraction (*f*_U_) determined in bicellar systems from MRE_215_, measured at 8 °C using CD, with increasing DMPC concentrations. *B* and *E,* Stern-Volmer constants (*K*_SV_) obtained from acrylamide quenching studies of the refolded proteins in DMPC:LDAO and DMPC:DDM at 25 °C are depicted, and compared with the aggregated protein (*Agg*). *C* and *F*, dependence of the *T_m_* of the refolded proteins to bicelle *q*, are shown for DMPC:LDAO and DMPC:DDM, and are derived from changes in MRE_215_. *A–F,* symbol/color schemes retained from Fig. 2*A. Error bars* depict the S.D. obtained from independent experiments. The data shown in *panels A–C* for refolded FL WT and FL C0 in DMPC:LDAO was originally published in Ref. 31, and has been reused here with permission, for comparison with the other constructs. *G* and *H,* differential scanning calorimetry profiles of empty DMPC:LDAO and DMPC:DDM bicelles, respectively, compared with pure DMPC vesicles (*purple line*).

We monitored hVDAC-2 behavior using CD, *T_m_*, and accessibility of tryptophans (*K*_SV_) (Fig. 7) (31). We obtained comparable refolding efficiency in all bicelle *q* from 0.00015 to 1.0, except for Δ^1–32^ WT, which failed to refold optimally at *q* >0.3 in DMPC:LDAO (20–30% higher unfolded fraction (*f*_U_) in Fig. 7, *A* and *D*). Microcalorimetry measurements (Fig. 7, *G* and *H*) reveal that at *q* > 0.3 (DMPC:LDAO) or *q* > 1 (DMPC:DDM) the bicelle phase transition changes from small isotropic bicelles (at low *q*) to bilayer-like character. Unassisted refolding of hVDAC-2 is, therefore, lower in membrane systems, and displays the order C0 > WT and FL > Δ^1–11^ > Δ^1–32^. NTE and NTH are therefore required for the hVDAC-2 barrel to refold in lipidic systems.

DDM supports hVDAC-2 refolding (compare Fig. 7, *A* and *D*) in bicelles of higher *q*, possibly because of its oblate micellar shape. Stronger protein-DMPC interactions in DMPC-DDM bicelles may also be responsible for this. Lower *K*_SV_ for the WT barrels in DMPC:LDAO and DMPC:DDM, as compared with the C0 constructs (Fig. 7, *B* and *E, left versus right panels*) suggests that indoles of the WT constructs are more lipid solvated. Hence, the strong barrel-micelle interaction exerted by the WT protein is also translated to lipid bicelles. The truncation mutants display low *K*_SV_ in conditions with poorer barrel refolding efficiency (Fig. 7, *B* and *E*). When we consider this data with the observed loss in secondary structure (*f*_U_ values in Fig. 7, *A* and *D*), the formation of a membrane-adsorbed barrel upon N-helix deletion is evident. As seen in micelles (Fig. 4*B*), NTE + NTH facilitate proper hVDAC-2 refolding in lipid bicelles.

Thermal stability measurements (Fig. 7, *C* and *F*) provide us with several interesting facets of hVDAC-2 behavior. (i) Similar *T_m_* values for all constructs across both bicelle systems suggest that our measurements correspond primarily to protein-DMPC interactions. (ii) The *T_m_* measured in micelles (*q* = 0) is retained in bicelles (*q* ≤ 0.015 for DMPC:LDAO and *q* ≤ 0.05 for DMPC:DDM) (Fig. 7, *C* and *F*). This indicates a structural similarity of hVDAC-2 between micelles and lipids, as seen for hVDAC-1 (43). (iii) In higher *q*, where the barrel exists in the adsorbed state, both WT and C0 barrels show comparable *T_m_*, although *f*_U_-WT < *f*_U_-C0 (compare Fig. 7, *A* and *C* or *D* and *F*). The adsorbed state of C0 constructs are, therefore, more β-rich. This is in good agreement with our previous observation that Cys-less constructs are well structured (32, 49). (iv) In DMPC:DDM bicelles, both the Δ^1–32^ constructs display a marginally higher *T_m_* value (Fig. 7*F*), although the *f*_U_ and *K*_SV_ values are similar to the FL and Δ^1–11^ barrels. The NTE + NTH-deleted hVDAC-2 may possibly tolerate higher lateral bilayer pressure in DMPC:DDM bicelles.

Our results clearly demonstrate that NTE and NTH are required for proper refolding of hVDAC-2 in lipidic systems. Once the correctly folded form is achieved, the stability of the barrel is primarily determined by the transmembrane β-sheets that form the pore. Most interestingly, the thermal stability dramatically decreases at high bicelle *q* (Fig. 7, *C* and *F*), suggesting that unlike hVDAC-1, hVDAC-2 requires additional stabilizing factors to sustain the bilayer pressure. In the mitochondrial membrane, these may include barrel oligomerization, interaction with lipids like cardiolipin or cholesterol, and binding to anti-apoptotic agents such as BAK.

## Discussion

The N-terminal helix of VDAC has attracted recent attention, due to its requirement for channel function and its involvement in several regulational processes, interaction with anti-apoptotic proteins, and cell survival (19, 20, 29). Additionally, the unique 11-residue sequence observed only in human isoform 2 is of intrigue from the structural and functional perspectives. However, studies on this isoform have been surprisingly limited. Our report is the first to embody the functional and biophysical requirement of the unique 11-residue NTE in hVDAC-2. We find that NTE is structured and plays an important role in the proper refolding of hVDAC-2 in both micelles and lipid bilayers. NTE suppresses the formation of undesirable off-pathway aggregates, and stabilizes the native state. Functionally, NTE confers asymmetric gating to the hVDAC-2 channel in DiPhPC membranes.

**FIGURE 8.**
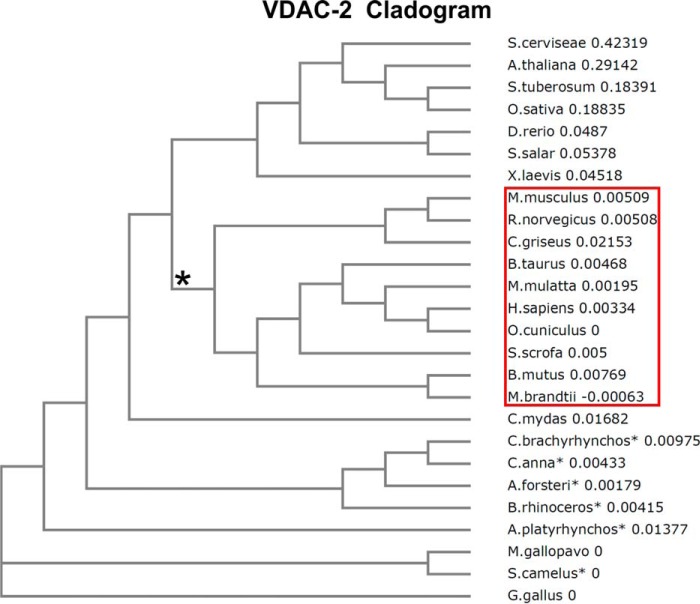
**Cladogram comparing sequence similarities in VDAC-2 across various organisms, created using ClustalW2 Phylogeny (58, 59).** The 10 organisms with a cysteine-rich VDAC-2 sequence that evolved from a common root (*) and show sequence conservation (see supplemental Fig. S1) are *boxed*. We believe that during sequence divergence, a Cys-rich barrel was selected earlier in evolution, after which the NTE was added to the primary sequence of VDAC-2. As a result, the conserved cysteines (Cys-133 and Cys-138, numbered according to hVDAC-2) are retained across all organisms that evolved from the root, but sequence similarity across the N-terminal residues is restricted to the higher mammals (see supplemental Fig. S1 for the alignment). Across different VDAC isoforms Cys-138 (numbered according to hVDAC-2) shows conservation in the first 10 organisms in both VDAC-1 and -2 (see supplemental Fig. S1 and S2), whereas Cys-133 (numbered according to hVDAC-2) is conserved in VDAC-2 and -3 (see supplemental Fig. S2 and S3).

NTE exerts an important influence on the barrel characteristics in the presence of cysteines. It is well known that the most abundant VDAC-1 isoform possesses only two cysteines; these cysteines are not important for apoptosis (52). However, hVDAC-2 has nine cysteines that orient toward the intermembrane space. Considering the sparsity of cysteines in proteins, the cysteine content of hVDAC-2 is unusually high. We have previously demonstrated that when cysteines are mutated, hVDAC-2 can exhibit characteristics akin to hVDAC-1 (31). NTE deletion in this Cys-less construct shows a less pronounced functional and biophysical manifestation compared with NTE-deleted hVDAC-2 WT. Our experimental observations allow us to conclude that the Cys-enriched WT barrel has specifically retained the 11-residue NTE for stability and function. Our hypothesis is additionally supported by the conservation of cysteines in VDAC-2, particularly in higher mammals that also possess NTE (Fig. 8 and supplemental Fig. S1). We are tempted to consider that in the course of evolution, VDAC-2 sequences of higher mammals, whereas gaining cysteines, may also have gained NTE to stabilize the barrel and as a means of incorporating more cysteines. Only two conserved cysteines are retained in all three isoforms and across most organisms (supplemental Figs. S1–S3), and these VDACs are devoid of NTE. NTE is therefore functionally unimportant for hVDAC-1, whereas it is evolutionarily significant in hVDAC-2.

VDACs are not the only known transmembrane β-barrels with a biologically important N-helix. The bacterial outer membrane enzyme PagP, for example, anchors itself to the phospholipid membrane through the N-terminal helix (53, 54). hVDAC-2 NTE(+NTH) serves the additional role of assisting proper refolding of the protein and in voltage sensing. Previous studies on hVDAC-1 have proposed a loss in barrel topology upon channel closure and deletion of NTH (7, 10, 48). The elliptical topology of the closed channel, seen upon removal of the N-helix, shows altered strand tilting (7, 48). Our experiments also demonstrate that externally supplemented NTH is unable to revive the voltage gating of hVDAC-2 Δ^1–32^. Could a similar elliptical topology therefore be accessible to hVDAC-2? The differential barrel-lipid interactions and structure-function studies we observe suggest that Δ^1–32^ C0 can access an elliptical closed state. In WT, on the other hand, our biophysical results support strong barrel-lipid interactions. Particularly, the observation that Δ^1–32^ WT mainly inserts in the open state as opposed to Δ^1–32^ C0 barrel, strongly advocates for a less collapsed topology in native hVDAC-2. Another interesting outcome of our study is the influence of cysteines on the barrel scaffold. Cysteines affect hVDAC-2 folding and the barrel tolerance to increased bilayer pressure when NTE + NTH are absent. Remarkably, the barrel-lipid interaction strengths are altered in the Δ^1–32^ C0 barrel, which could stem from the elliptical conformation. Such interactions depend on whether the micelle packing can accommodate the altered topology. LDAO experiences electrostatic repulsion in the headgroup (55) and are more suited to stabilize spherical hVDAC-2 structures that impose lesser demands on micelle packing. The non-ionic oblate DDM micelles have lesser hydrophobic diameter and do not suffer from packing defects as LDAO (55). DDM can therefore promote protein-micelle interactions in both spherical and elliptical barrel forms. This feature of DDM is possibly why we obtain DMPC:DDM bicelles of higher *q* and optimal lateral pressure for refolding hVDAC-2.

In hVDAC-2, our experiments in 12-C micellar systems translate reproducibly to the 14-C lipidic bicelles. A similar structural scaffold is therefore retained by this protein in various systems. Furthermore, the contribution of NTE and NTH are comparable in LDAO, DDM and DMPC. Our measurements, therefore, correspond primarily to the interaction of the N-helix with the β-barrel, and the β-barrel with its lipid environment. The intrinsic amino acid composition, secondary structure, and tertiary interactions mediated through NTH and NTE together contribute substantially to the overall hVDAC-2 stability. hVDAC-2 topology and strand tilt can be altered by the lipid environment (7, 48), in a cysteine- and N-helix dependent manner, through likely changes in the shear number. The loss in barrel stability with an increase in bilayer lateral pressure suggests that agents such as unsaturated lipids, which introduce defects in ordered lipids, promote refolding and control residence time of the barrel in the mitochondrial outer membrane.

Our observation that hVDAC-2 can remain in the “open” state even in the absence of the N-helix opens new avenues for its contribution to apoptosis. The role of cysteines of hVDAC-2 and hVDAC-3 in ROS regulation has been speculated for some time. Irreversible cysteine oxidation during ROS build-up can undock the N-helix from within the barrel. As hVDAC-2 can retain its open state in this condition, it is tempting to speculate that this conformation can ultimately lead to unregulated mitochondrial outer membrane permeabilization and cell death. Furthermore, conformational changes imposed by lateral bilayer pressure can alter the barrel topology *in vivo* and affect N-helix interaction with the Bcl-2 protein family. Different barrel interaction surfaces thus presented to the Bcl-2 proteins can reflect the antagonistic apoptotic functions of hVDAC-1 and hVDAC-2 (6, 14, 25, 26). Our study delivers new insight on barrel-micelle, barrel-lipid interactions, which, when taken together with hVDAC-2 cysteines and the N-terminal region, can control barrel dynamics. Such conformational changes imposed by lateral bilayer pressure and N-helix interaction can cause structural changes and lead to different barrel stability. As has been recently seen for BAX-mediated apoptosis (56), this process can trigger or halt the apoptotic pathway.

## Author Contributions

R. M. conceived the study. S. R. M. performed the experiments. Both authors analyzed the data and wrote the paper.

## Supplementary Material

Supplemental Data

## Footnotes

3 The abbreviations used are:

hVDAC: human voltage-dependent anion channel
NTH: N-terminal helix
NTE: N-terminal extension
ROS: reactive oxygen species
Δ^1–32^: hVDAC-2 with NTH deletion
Δ^1–11^: hVDAC-2 with NTE deletion
WT: wild-type hVDAC-2 protein
C0: Cys-less mutant of hVDAC-2
FL: full-length
LDAO: lauryldimethylamine oxide
DDM: dodecyl β-d-maltoside
GdnHCl: guanidine hydrochloride
DMPC: 1,2-dimyristoyl-*sn*-glycero-3-phosphocholine
q: long chain lipid/short chain lipid
DiPhPC: diphytanoyl phosphatidylcholine
*r*: anisotropy
*f*_U_: unfolded fraction
*f*_F_: folded fraction
Δ*G*_U_^0^: Gibbs free energy of unfolding
Δ*G*_app_^0^: apparent Gibbs free energy of unfolding
*m*_app_: apparent unfolding cooperativity
*C_m_*: chemical denaturation mid-point
*K*_SV_: Stern-Volmer quenching constant
*k_q_*: bimolecular quenching constant
τ*_c_*: rotational correlation time
CD: circular dichroism
MRE_215_: mean residue ellipticity at 215 nm
*T_m_*: thermal denaturation mid-point
*T*_*m*-start_: start temperature of thermal denaturation.

## References

[1] Messina A., Reina S., Guarino F., and De Pinto V. (2012) VDAC isoforms in mammals. Biochim. Biophys. Acta 1818, 1466–1476 22020053 10.1016/j.bbamem.2011.10.005

[2] Shoshan-Barmatz V., Ben-Hail D., Admoni L., Krelin Y., and Tripathi S. S. (2015) The mitochondrial voltage-dependent anion channel 1 in tumor cells. Biochim. Biophys. Acta 1848, 2547–2575 25448878 10.1016/j.bbamem.2014.10.040

[3] Shoshan-Barmatz V., De Pinto V., Zweckstetter M., Raviv Z., Keinan N., and Arbel N. (2010) VDAC, a multi-functional mitochondrial protein regulating cell life and death. Mol. Aspects Med. 31, 227–285 20346371 10.1016/j.mam.2010.03.002

[4] Bayrhuber M., Meins T., Habeck M., Becker S., Giller K., Villinger S., Vonrhein C., Griesinger C., Zweckstetter M., and Zeth K. (2008) Structure of the human voltage-dependent anion channel. Proc. Natl. Acad. Sci. U.S.A. 105, 15370–15375 18832158 10.1073/pnas.0808115105 PMC2557026

[5] Hiller S., Garces R. G., Malia T. J., Orekhov V. Y., Colombini M., and Wagner G. (2008) Solution structure of the integral human membrane protein VDAC-1 in detergent micelles. Science 321, 1206–1210 18755977 10.1126/science.1161302 PMC2579273

[6] Ujwal R., Cascio D., Colletier J. P., Faham S., Zhang J., Toro L., Ping P., and Abramson J. (2008) The crystal structure of mouse VDAC1 at 2.3-Å resolution reveals mechanistic insights into metabolite gating. Proc. Natl. Acad. Sci. U.S.A. 105, 17742–17747 18988731 10.1073/pnas.0809634105 PMC2584669

[7] Zachariae U., Schneider R., Briones R., Gattin Z., Demers J. P., Giller K., Maier E., Zweckstetter M., Griesinger C., Becker S., Benz R., de Groot B. L., and Lange A. (2012) β-Barrel mobility underlies closure of the voltage-dependent anion channel. Structure 20, 1540–1549 22841291 10.1016/j.str.2012.06.015 PMC5650048

[8] Cheng E. H., Sheiko T. V., Fisher J. K., Craigen W. J., and Korsmeyer S. J. (2003) VDAC2 inhibits BAK activation and mitochondrial apoptosis. Science 301, 513–517 12881569 10.1126/science.1083995

[9] Schredelseker J., Paz A., López C. J., Altenbach C., Leung C. S., Drexler M. K., Chen J. N., Hubbell W. L., and Abramson J. (2014) High resolution structure and double electron-electron resonance of the zebrafish voltage-dependent anion channel 2 reveal an oligomeric population. J. Biol. Chem. 289, 12566–12577 24627492 10.1074/jbc.M113.497438 PMC4007448

[10] Schneider R., Etzkorn M., Giller K., Daebel V., Eisfeld J., Zweckstetter M., Griesinger C., Becker S., and Lange A. (2010) The native conformation of the human VDAC1 N terminus. Angew. Chem. Int. Ed. Engl. 49, 1882–1885 20140924 10.1002/anie.200906241

[11] Geula S., Ben-Hail D., and Shoshan-Barmatz V. (2012) Structure-based analysis of VDAC1: N-terminus location, translocation, channel gating and association with anti-apoptotic proteins. Biochem. J. 444, 475–485 22397371 10.1042/BJ20112079

[12] Guo X. W., Smith P. R., Cognon B., D'Arcangelis D., Dolginova E., and Mannella C. A. (1995) Molecular design of the voltage-dependent, anion-selective channel in the mitochondrial outer membrane. J. Struct. Biol. 114, 41–59 7772417 10.1006/jsbi.1995.1004

[13] Stanley S., Dias J. A., D'Arcangelis D., and Mannella C. A. (1995) Peptide-specific antibodies as probes of the topography of the voltage-gated channel in the mitochondrial outer membrane of *Neurospora crassa*. J. Biol. Chem. 270, 16694–16700 7542652 10.1074/jbc.270.28.16694

[14] Blachly-Dyson E., Peng S., Colombini M., and Forte M. (1990) Selectivity changes in site-directed mutants of the VDAC ion channel: structural implications. Science 247, 1233–1236 1690454 10.1126/science.1690454

[15] Song J., Midson C., Blachly-Dyson E., Forte M., and Colombini M. (1998) The topology of VDAC as probed by biotin modification. J. Biol. Chem. 273, 24406–24413 9733730 10.1074/jbc.273.38.24406

[16] Reymann S., Flörke H., Heiden M., Jakob C., Stadtmüller U., Steinacker P., Lalk V. E., Pardowitz I., and Thinnes F. P. (1995) Further evidence for multitopological localization of mammalian porin (VDAC) in the plasmalemma forming part of a chloride channel complex affected in cystic fibrosis and encephalomyopathy. Biochem. Mol. Med. 54, 75–87 8581362 10.1006/bmme.1995.1011

[17] De Pinto V., Tomasello F., Messina A., Guarino F., Benz R., La Mendola D., Magrì A., Milardi D., and Pappalardo G. (2007) Determination of the conformation of the human VDAC1 N-terminal peptide, a protein moiety essential for the functional properties of the pore. Chem. Bio. Chem. 8, 744–756 10.1002/cbic.200700009 17387661

[18] Tewari D., Ahmed T., Chirasani V. R., Singh P. K., Maji S. K., Senapati S., and Bera A. K. (2015) Modulation of the mitochondrial voltage dependent anion channel (VDAC) by curcumin. Biochim. Biophys. Acta 1848, 151–158 25459681 10.1016/j.bbamem.2014.10.014

[19] Guardiani C., Scorciapino M. A., Amodeo G. F., Grdadolnik J., Pappalardo G., De Pinto V., Ceccarelli M., and Casu M. (2015) The N-terminal peptides of the three human isoforms of the mitochondrial voltage-dependent anion channel have different helical propensities. Biochemistry 54, 5646–5656 26303511 10.1021/acs.biochem.5b00469

[20] Abu-Hamad S., Arbel N., Calo D., Arzoine L., Israelson A., Keinan N., Ben-Romano R., Friedman O., and Shoshan-Barmatz V. (2009) The VDAC1 N-terminus is essential both for apoptosis and the protective effect of anti-apoptotic proteins. J. Cell Sci. 122, 1906–1916 19461077 10.1242/jcs.040188

[21] Popp B., Court D. A., Benz R., Neupert W., and Lill R. (1996) The role of the N and C termini of recombinant *Neurospora* mitochondrial porin in channel formation and voltage-dependent gating. J. Biol. Chem. 271, 13593–13599 8662769 10.1074/jbc.271.23.13593

[22] De Pinto V., Reina S., Guarino F., and Messina A. (2008) Structure of the voltage dependent anion channel: state of the art. J. Bioenerg. Biomembr. 40, 139–147 18668358 10.1007/s10863-008-9140-3

[23] Mertins B., Psakis G., Grosse W., Back K. C., Salisowski A., Reiss P., Koert U., and Essen L. O. (2012) Flexibility of the N-terminal mVDAC1 segment controls the channel's gating behavior. PLoS ONE 7, e47938 23110136 10.1371/journal.pone.0047938 PMC3479125

[24] Gattin Z., Schneider R., Laukat Y., Giller K., Maier E., Zweckstetter M., Griesinger C., Benz R., Becker S., and Lange A. (2015) Solid-state NMR, electrophysiology and molecular dynamics characterization of human VDAC2. J. Biomol. NMR 61, 311–320 25399320 10.1007/s10858-014-9876-5 PMC5653203

[25] Mannella C. A. (1990) Structural analysis of mitochondrial pores. Experientia 46, 137–145 1689251 10.1007/BF02027309

[26] Zimmerberg J., and Parsegian V. A. (1986) Polymer inaccessible volume changes during opening and closing of a voltage-dependent ionic channel. Nature 323, 36–39 2427958 10.1038/323036a0

[27] Peng S., Blachly-Dyson E., Forte M., and Colombini M. (1992) Large scale rearrangement of protein domains is associated with voltage gating of the VDAC channel. Biophys. J. 62, 123–131 1376163 10.1016/S0006-3495(92)81799-X PMC1260505

[28] Teijido O., Ujwal R., Hillerdal C. O., Kullman L., Rostovtseva T. K., and Abramson J. (2012) Affixing N-terminal α-helix to the wall of the voltage-dependent anion channel does not prevent its voltage gating. J. Biol. Chem. 287, 11437–11445 22275367 10.1074/jbc.M111.314229 PMC3322836

[29] Reina S., Palermo V., Guarnera A., Guarino F., Messina A., Mazzoni C., and De Pinto V. (2010) Swapping of the N-terminus of VDAC1 with VDAC3 restores full activity of the channel and confers anti-aging features to the cell. FEBS Lett. 584, 2837–2844 20434446 10.1016/j.febslet.2010.04.066

[30] Bauer A. J., Gieschler S., Lemberg K. M., McDermott A. E., and Stockwell B. R. (2011) Functional model of metabolite gating by human voltage-dependent anion channel 2. Biochemistry 50, 3408–3410 21425834 10.1021/bi2003247 PMC3082971

[31] Maurya S. R., and Mahalakshmi R. (2013) Modulation of human mitochondrial voltage-dependent anion channel 2 (hVDAC-2) structural stability by cysteine-assisted barrel-lipid interactions. J. Biol. Chem. 288, 25584–25592 23873934 10.1074/jbc.M113.493692 PMC3757219

[32] Maurya S. R., and Mahalakshmi R. (2014) Cysteine residues impact the stability and micelle interaction dynamics of the human mitochondrial β-barrel anion channel hVDAC-2. PLoS ONE 9, e92183 24642864 10.1371/journal.pone.0092183 PMC3967697

[33] Liu M. Y., and Colombini M. (1992) A soluble mitochondrial protein increases the voltage dependence of the mitochondrial channel, VDAC. J. Bioenerg. Biomembr. 24, 41–46 1380504 10.1007/BF00769529

[34] Makwana K. M., Raghothama S., and Mahalakshmi R. (2013) Stabilizing effect of electrostatic *vs.* aromatic interactions in diproline nucleated peptide β-hairpins. Phys. Chem. Chem. Phys. 15, 15321–15324 23942893 10.1039/c3cp52770k

[35] Iyer B. R., and Mahalakshmi R. (2015) Residue-dependent thermodynamic cost and barrel plasticity balances activity in the PhoPQ-activated enzyme PagP of *Salmonella typhimurium*. Biochemistry 54, 5712–5722 26334694 10.1021/acs.biochem.5b00543

[36] Pocanschi C. L., Popot J. L., and Kleinschmidt J. H. (2013) Folding and stability of outer membrane protein A (OmpA) from *Escherichia coli* in an amphipathic polymer, amphipol A8–35. Eur. Biophys. J. 42, 103–118 23370791 10.1007/s00249-013-0887-z

[37] Greenfield N. J. (2006) Using circular dichroism collected as a function of temperature to determine the thermodynamics of protein unfolding and binding interactions. Nat. Protoc. 1, 2527–2535 17406506 10.1038/nprot.2006.204 PMC2752288

[38] Teijido O., Rappaport S. M., Chamberlin A., Noskov S. Y., Aguilella V. M., Rostovtseva T. K., and Bezrukov S. M. (2014) Acidification asymmetrically affects voltage-dependent anion channel implicating the involvement of salt bridges. J. Biol. Chem. 289, 23670–23682 24962576 10.1074/jbc.M114.576314 PMC4156087

[39] Rostovtseva T. K., Kazemi N., Weinrich M., and Bezrukov S. M. (2006) Voltage gating of VDAC is regulated by nonlamellar lipids of mitochondrial membranes. J. Biol. Chem. 281, 37496–37506 16990283 10.1074/jbc.M602548200

[40] Reina S., Magrì A., Lolicato M., Guarino F., Impellizzeri A., Maier E., Benz R., Ceccarelli M., De Pinto V., and Messina A. (2013) Deletion of β-strands 9 and 10 converts VDAC1 voltage-dependence in an asymmetrical process. Biochim. Biophys. Acta 1827, 793–805 23541892 10.1016/j.bbabio.2013.03.007

[41] Andrews B. T., Capraro D. T., Sulkowska J. I., Onuchic J. N., and Jennings P. A. (2013) Hysteresis as a marker for complex, overlapping landscapes in proteins. J. Phys. Chem. Lett. 4, 180–188 23525263 10.1021/jz301893w PMC3601837

[42] Oliver R. C., Lipfert J., Fox D. A., Lo R. H., Doniach S., and Columbus L. (2013) Dependence of micelle size and shape on detergent alkyl chain length and head group. PLoS ONE 8, e62488 23667481 10.1371/journal.pone.0062488 PMC3648574

[43] Eddy M. T., Su Y., Silvers R., Andreas L., Clark L., Wagner G., Pintacuda G., Emsley L., and Griffin R. G. (2015) Lipid bilayer-bound conformation of an integral membrane β barrel protein by multidimensional MAS NMR. J. Biomol. NMR 61, 299–310 25634301 10.1007/s10858-015-9903-1 PMC4398622

[44] Privé G. G. (2007) Detergents for the stabilization and crystallization of membrane proteins. Methods 41, 388–397 17367711 10.1016/j.ymeth.2007.01.007

[45] Moon C. P., Kwon S., and Fleming K. G. (2011) Overcoming hysteresis to attain reversible equilibrium folding for outer membrane phospholipase A in phospholipid bilayers. J. Mol. Biol. 413, 484–494 21888919 10.1016/j.jmb.2011.08.041 PMC3193555

[46] Wimley W. C., and White S. H. (1996) Experimentally determined hydrophobicity scale for proteins at membrane interfaces. Nat. Struct. Biol. 3, 842–848 8836100 10.1038/nsb1096-842

[47] Moon C. P., Zaccai N. R., Fleming P. J., Gessmann D., and Fleming K. G. (2013) Membrane protein thermodynamic stability may serve as the energy sink for sorting in the periplasm. Proc. Natl. Acad. Sci. U.S.A. 110, 4285–4290 23440211 10.1073/pnas.1212527110 PMC3600475

[48] Kozuch J., Weichbrodt C., Millo D., Giller K., Becker S., Hildebrandt P., and Steinem C. (2014) Voltage-dependent structural changes of the membrane-bound anion channel hVDAC1 probed by SEIRA and electrochemical impedance spectroscopy. Phys. Chem. Chem. Phys. 16, 9546–9555 24728177 10.1039/c4cp00167b

[49] Maurya S. R., and Mahalakshmi R. (2014) Influence of protein- micelle ratios and cysteine residues on the kinetic stability and unfolding rates of human mitochondrial VDAC-2. PLoS ONE 9, e87701 24494036 10.1371/journal.pone.0087701 PMC3907894

[50] Minetti C. A., and Remeta D. P. (2006) Energetics of membrane protein folding and stability. Arch. Biochem. Biophys. 453, 32–53 16712771 10.1016/j.abb.2006.03.023

[51] Wimley W. C., and White S. H. (2004) Reversible unfolding of β-sheets in membranes: a calorimetric study. J. Mol. Biol. 342, 703–711 15342231 10.1016/j.jmb.2004.06.093 PMC2935845

[52] Aram L., Geula S., Arbel N., and Shoshan-Barmatz V. (2010) VDAC1 cysteine residues: topology and function in channel activity and apoptosis. Biochem. J. 427, 445–454 20192921 10.1042/BJ20091690

[53] Ahn V. E., Lo E. I., Engel C. K., Chen L., Hwang P. M., Kay L. E., Bishop R. E., and Privé G. G. (2004) A hydrocarbon ruler measures palmitate in the enzymatic acylation of endotoxin. EMBO J. 23, 2931–2941 15272304 10.1038/sj.emboj.7600320 PMC514935

[54] Huysmans G. H., Radford S. E., Brockwell D. J., and Baldwin S. A. (2007) The N-terminal helix is a post-assembly clamp in the bacterial outer membrane protein PagP. J. Mol. Biol. 373, 529–540 17868697 10.1016/j.jmb.2007.07.072 PMC2887491

[55] Kaufmann T. C., Engel A., and Rémigy H. W. (2006) A novel method for detergent concentration determination. Biophys. J. 90, 310–317 16214861 10.1529/biophysj.105.070193 PMC1367029

[56] Renault T. T., Floros K. V., Elkholi R., Corrigan K. A., Kushnareva Y., Wieder S. Y., Lindtner C., Serasinghe M. N., Asciolla J. J., Buettner C., Newmeyer D. D., and Chipuk J. E. (2015) Mitochondrial shape governs BAX-induced membrane permeabilization and apoptosis. Mol. Cell 57, 69–82 25482509 10.1016/j.molcel.2014.10.028 PMC4289414

[57] Roy A., Kucukural A., and Zhang Y. (2010) I-TASSER: a unified platform for automated protein structure and function prediction. Nat. Protoc. 5, 725–738 20360767 10.1038/nprot.2010.5 PMC2849174

[58] Larkin M. A., Blackshields G., Brown N. P., Chenna R., McGettigan P. A., McWilliam H., Valentin F., Wallace I. M., Wilm A., Lopez R., Thompson J. D., Gibson T. J., and Higgins D. G. (2007) Clustal W and Clustal X version 2.0. Bioinformatics 23, 2947–2948 17846036 10.1093/bioinformatics/btm404

[59] Goujon M., McWilliam H., Li W., Valentin F., Squizzato S., Paern J., and Lopez R. (2010) A new bioinformatics analysis tools framework at EMBL-EBI. Nucleic Acids Res. 38, W695–W699 20439314 10.1093/nar/gkq313 PMC2896090

