# Supplementary material for: N-helix and Cysteines Inter-regulate Human Mitochondrial VDAC-2 Function and Biochemistry
Source: J Biol Chem. 2015 Oct 20;290(51):30240–52. doi: 10.1074/jbc.M115.693978 (PMC4683249; doi:10.1074/jbc.M115.693978)
Supplement: Supplemental Data [file supp_290_51_30240__index.html]

N-helix and cysteines inter-regulate human mitochondrial VDAC-2 function and biochemistry — N-helix and Cysteines Inter-regulate Human Mitochondrial VDAC-2 Function and Biochemistry — Molecular Regulators of hVDAC-2 Stability and Function — Supplemental Data 

# N-helix and Cysteines Inter-regulate Human Mitochondrial VDAC-2 Function and Biochemistry

## Supplemental Data

- Supplemental Fig. (.pdf, 697 KB) - Multiple sequence alignments.
