# Supplementary material for: N-helix and Cysteines Inter-regulate Human Mitochondrial VDAC-2 Function and Biochemistry
Source: J Biol Chem. 2015 Oct 20;290(51):30240–52. doi: 10.1074/jbc.M115.693978 (PMC4683249; doi:10.1074/jbc.M115.693978)
Supplement: Supplemental Data [file 10.1074_M115.693978_jbc.M115.693978-1.pdf]

N-helix and cysteines inter-regulate human mitochondrial VDAC-2 function and biochemistry

Svetlana Rajkumar Maurya and Radhakrishnan Mahalakshmi[1]

Molecular Biophysics Laboratory, Department of Biological Sciences,
Indian Institute of Science Education and Research, Bhopal 462023. India.

[1]To whom correspondence should be addressed: Dr. R. Mahalakshmi, Department of Biological Sciences, Indian Institute of Science Education and Research, Transit campus: ITI Govindpura, Bhopal 462023. India. Tel: +91-755-4092318; Fax: +91-755-4092392; E-mail: maha@iiserb.ac.in.

SUPPLEMENTAL FIGURES

```
                             10        20        30        40        50        60        70        80        90       100       110
                    ....|....|....|....|....|....|....|....|....|....|....|....|....|....|....|....|....|....|....|....|....|....|
H.sapiens           ------------MATHGQTCARPMCIPPSYADLGKAARDIFNKGFGF-GLVKLDVKTKSCSGVEFSTSGSSNTDTGKVTGTLETKYKWCEYGLTFTEKWNTDNTLGTEIA
C.griseus           ------------MLTIECFFLLAMCIPPCYADLGKAARDIFNKGFGF-GLVKLDVKTKSCSGVEFSTSGSSNTDTGKVSGTLETKYKWCEYGLTFTEKWNTDNTLGTEIA
B.taurus            ------------MATHGQNCARPMCIPPSYADLGKAARDIFNKGFGF-GLVKLDVKTKSCSGVEFSTSGSSNTDTGKVTGTLETKYKWCEYGLTFTEKWNTDNTLGTEIA
S.scrofa            ------------MATHGQTCPRPMCIPPSYADLGKAARDIFNKGFGF-GLVKLDVKTKSCSGVEFSTSGSSNTDTGKVTGTLETKYKWCEYGLTFTEKWNTDNTLGTEIA
M.mulatta           ------------MATYGQTCARPMCIPPSYADLGKAARDIFNKGFGF-GLVKLDVKTKSCSGVEFSTSGSSNTDTGKVTGTLETKYKWCEYGLTFTEKWNTDNTLGTEIA
O.cuniculus         ------------MATHGQTCARPMCIPPSYADLGKAARDIFNKGFGF-GLVKLDVKTKSCSGVEFSTSGSSNTDTGKVTGTLETKYKWCEYGLTFTEKWNTDNTLGTEIA
M.musculus          -----------MAECCVPVCPRPMCIPPPYADLGKAARDIFNKGFGF-GLVKLDVKTKSCSGVEFSTSGSSNTDTGKVSGTLETKYKWCEYGLTFTEKWNTDNTLGTEIA
R.norvegicus        -----------MAECCVPVCQRPICIPPPYADLGKAARDIFNKGFGF-GLVKLDVKTKSCSGVEFSTSGSSNTDTGKVSGTLETKYKWCEYGLTFTEKWNTDNTLGTEIA
B.mutus             MSWCGELRLLALKPHSIGRGLESMCIPPSYADLGKAARDIFNKGFGF-GLVKLDVKTKSCSGVEFSTSGSSNTDTGKVTGTLETKYKWCEYGLTFTEKWNTDNTLGTEIA
M.brandtii          -----------------------MCIPPSYADLGKAARDIFNKGFGF-GLVKLDVKTKSCSGVEFSTSGSSNTDTGKVTGTLETKYKWCEYGLTFTEKWNTDNTLGTEIA
X.laevis            -----------------------MAVPPSYADLGKSARDIFNKGYGF-GLVKLDVKTKSATGVEFTTSGTSNTDSGKVNGSLETKYKWGEYGLTFTEKWNTDNTLGTEIA
C.mydas             -----------------------MAIPPSYADLGKSARDIFNKGYA----------------------GSSNTDTGKVNGSLETKYKWAEYGLTFTEKWNTDNTLGTEIA
A.platyrhynchos*    ----------------------PMAIPPSYADLGKSARDIFNKGYGF-GLVKLDVKTKSASGVEFTTSGSSNTDTGKVNGSLETKYKWAEYGLTFTEKRNTDNTLGTEIA
C.brachyrhynchos*   ----------------------PMAIPPSYVDLGKPARDIFNKGYGF-GLVKLDVKTKSASGVEFTTSGSSNTDTGKVNGSLETKYKWAEYGLTFTEKWNTDNTLGTEIA
C.anna*             ----------------------PMAIPPSYVDLGKSARDIFNKGYGF-GLVKLDVKTKSATGVEFTTSGSSNTDTGKVNGSLETKYKWAEYGLTFTEKWNTDNTLGTEIA
B.rhinoceros*       ----------------------PMAIPPSYVDLGKSARDIFNKGYGF-GLVKLDVKTKSASGVEFTTSGSSNTDTGKVNGSLETKYKWAEYGLTFTEKWNTDNTLGTEIA
A.forsteri*         ----------------------PMAIPPSYVDLGKSARDIFNKGYGF-GLVKLDVKTKSASGVEFTTSGSSNTDTGKVNGSLETKYKWAEYGLTFTEKWNTDNTLGTEIA
S.camelus*          ----------------------PMAIPPSYADLGKSARDIFNKGYGF-GLVKLDVKTKSASGVEFTTSGSSNTDTGKVNGSLETKYKWAEYGLTFTEKWNTDNTLGTEIA
M.gallopavo         ------------------------AIPPSYADLGKSARDIFNKGYGF-GLVKLDVKTKSASGVEFTTSGSSNTDTGKVNGSLETKYKWAEYGLTFTEKWNTDNTLGTEIA
G.gallus            -----------------------MAIPPSYADLGKSARDIFNKGYGF-GLVKLDVKTKSASGVEFTTSGSSNTDTGKVNGSLETKYKWAEYGLTFTEKWNTDNTLGTEIA
D.rerio             -----------------------MAVPPAYADLGKSAKDIFNKGYGF-GMVKLDVKTKSASGVEFKTSGSSNTDTSKVVGSLETKYKRSEYGLTFTEKWNTDNTLGTEIN
S.salar             -----------------------MAVPPSYGDLGKSAKDIFNKGYGF-GLVKLDVKTKSSSGVEFKTSGSSNTDTSKVNGNLETKYKWAEYGLTFTEKWTTDNTLGTEIT
A.thaliana          ----------------------MSKGPGLFTDIGKKAKDLLTRDYNS-D-QKFSISTYSASGVALTSTALKKGGVH--AADVATQYKY--KNALFDVKIDTDSSVLTTVT
S.tuberosum         ----------------------MVKGPGLYSDIGKKARDLLYRDYVS-D-HKFTVTTYSTTGVAITASGLKKGELF--LADVSTQLKN--KNITTDVKVDTNSNVYTTIT
O.sativa            -----------------MAAAAPPAGPGLYSDIGKKARDLLYRDYHT-D-QKFTLTTYAANGAAITVAGTKKNESI--FSEIQSQVKN--NNVSVDVKATSDSKLITTFT
S.cerviseae         ------------------------MALRFFNDISRDVNGLFNRDFFHTNPLSLNISTTTENGVNFTLKAKQGVTEGPIQTSVEGRFYDRKEGVSLSQSWSNQNRLNTRIE
Clustal Consensus                               : *:.: .. :: : :                         . .            :  :              .  .:. : * .

                            120       130       140       150       160       170       180       190       200       210       220
                    ....|....|....|....|....|....|....|....|....|....|....|....|....|....|....|....|....|....|....|....|....|....|
H.sapiens           IEDQICQGLKLTFDTTFSPNTGKKSGKIKSSYKRECINLGCDVDFDFAGPAIHGSAVFGYEGWLAGYQMTFDSAKSKLTRNNFAVGYRTGDFQLHTNVND-GTEFGGSIY
C.griseus           IEDQICQGLKLTFDTTFSPNTGKKSGKIKSAYKRECINLGCDVDFDFAGPAIHGSAVFGYEGWLAGYQMTFDSAKSKLTRNNFAVGYRTGDFQLHTNVNN-GTEFGGSIY
B.taurus            IEDQICQGLKLTFDTTFSPNTGKKSGKIKSSYKRECINLGCDVDFDFAGPAIHGSAVFGYEGWLAGYQMTFDSAKSKLTRNNFAVGYRTGDFQLHTNVND-GTEFGGSIY
S.scrofa            IEDQICQGLKLTFDTTFSPNTGKKSGKIKSSYKRECVNLGCDVDFDFAGPAIHGSAVFGYEGWLAGYQMTFDSAKSKLTRNNFAVGYRTGDFQLHTNVND-GTEFGGSIY
M.mulatta           IEDQICQGLKLTFDTTFSPNTGKKSGKIKSSYKRECINLGCDVDFDFAGPAIHGSAVFGYEGWLAGYQMTFDSAKSKLTRNNFAVGYRTGDFQLHTNVND-GTEFGGSIY
O.cuniculus         IEDQICQGLKLTFDTTFSPNTGKKSGKIKSSYKRECINLGCDVDFDFAGPAIHGSAVFGYEGWLAGYQMTFDSAKSKLTRNNFAVGYRTGDFQLHTNVND-GTEFGGSIY
M.musculus          IEDQICQGLKLTFDTTFSPNTGKKSGKIKSAYKRECINLGCDVDFDFAGPAIHGSAVFGYEGWLAGYQMTFDSAKSKLTRSNFAVGYRTGDFQLHTNVNN-GTEFGGSIY
R.norvegicus        IEDQICQGLKLTFDTTFSPNTGKKSGKIKSAYKRECINLGCDVDFDFAGPAIHGSAVFGYEGWLAGYQMTFDSAKSKLTRSNFAVGYRTGDFQLHTNVNN-GTEFGGSIY
B.mutus             IEDQICQGLKLTFDTTFSPNTGKKSGKIKSSYKRECINLGCDVDFDFAGPAIHGSAVFGYEGWLAGYQMTFDSAKSKLTRNNFAVGYRTGDFQLHTNVND-GTEFGGSIY
M.brandtii          IEDQICQGLKLTFDTTFSPNTGKKSGKIKSSYKREWINLGCDTDFDFAGPAIHGSAVFGYEGWLAGYQMTFDSAKSKLTRNNFAVGYRTGDFQLHTNVND-GTEFGGSIY
X.laevis            IEDQIAKGLKLTFDTTFSPNTGKKSGKVKAAYKQEYVNLGCDVDFDFAGPAIHGSAVVGYEGWLAGYQMTFDSAKSKLTKNNFAVGYKTGDFQLHTNVND-GSEFAGSIY
C.mydas             IEDQIAKGLKLTFDTTFSPNTGKKSGKIKSSYKRECVNLGCDVDFDFAGPAIHGSAVVGYEGWLAGYQMTFDSAKSKLTRNNFSVGYKTGDFQLHTNVND-GSEFGGSIY
A.platyrhynchos*    IEDQIAKGLKLTFDTTFSPNTGKKSGKIKSAYKRECLNLGCDVDFDFAGPAIHGSAVFGYEGWLAGYQMTFDSAKSKLTRNNFSVCYKTGDFQLHTNVND-GSEFGGSIY
C.brachyrhynchos*   IEDQIAKGLKLTFDTTFSPNTGKKSGKIKSAYKRECVNLGCDVDFDFAGPAIHGSAVFGFEGWLAGYQMTFDSAKSKLTRNNFSVGYKTGDFQLHTNVND-GSEFGGSIY
C.anna*             IEDQIAKGLKLTFDTTFSPNTGKKSGKIKSAYKRECLNLGCDVDFDFAGPAIHGSAVFGYEGWLAGYQMTFDSAKSKLTRNNFSVGYKTGDFQLHTNVND-GSEFGGSIY
B.rhinoceros*       IEDQIAKGLKLTFDTTFSPNTGKKSGKIKSAYKRECLNIGCDVDFDFAGPAIHGSAVFGYEGWLAGYQMTFDSAKSKLTRNNFSVGYKTGDFQLHTNVND-GSEFGGSIY
A.forsteri*         IEDQIAKGLKLTFDTTFSPNTGKKSGKIKSAYKRECVNLGCDVDFDFAGPAIHGSAVFGYEGWLAGYQMTFDSAKSKLTRNNFSVGYKTGDFQLHTNVND-GSEFGGSIY
S.camelus*          IEDQIAKGLKLTFDTTFSPNTGKKSGKIKSAYKRECLNLGCDVDFDFAGPAIHGSAVFGYEGWLAGYQMTFDSAKSKLTRNNFSVGYKTGDFQLHTNVND-GSEFGGSIY
```

```
M.gallopavo          IEDQIAKGLKLTFDTTFSPNTGKKSGKIKSAYKRECLNLGCDVDFDFAGPAIHGSAVFGYEGWLAGYQMTFDSAKSKLTRNNFSVGYKTGDFQLHTNVND-GSEFGGSIY
G.gallus             IEDQIAKGLKLTFDTTFSPNTGKKSGKIKSAYKRECLNLGCDVDFDFAGPAIHGSAVFGYEGWLAGYQMTFDSAKSKLTRNNFSVGYKTGDFQLHTNVND-GSEFGGSIY
D.rerio              IEDQIAKGLKLTFDTTFSPNTGKKSGKVKTAYKREFVNLGCDVDFDFAGPTIHGAAVVGYEGWLAGYQMSFDTAKSKMTQNNFAVGYKTGDFQLHTNVND-GSEFGGSIY
S.salar              VEDQITKGLKLTFDTQFSPNSGKKSGKVKTAYKREYVNLGVDVDFDFAGPAIHGAAVAGYEGWLAGYQMTFDTAKSKMTQSNFAVGYKTGDFQLHTNVND-GTEFGGSIY
A.thaliana           LT-EILPSTKAIASFKVP---DYNSAKLEVQYFHDHATVTAAAALK-QNPLIDITATLGSPVISFGAEAGYDTTSKTFTKYNAGISVTKPDACLSIILGDKGDSLKASYL
S.tuberosum          VD-EPAPGLKTIFSFVVP---DQKSGKVELQYLHEYAGINTSIGLT-ASPLVNFSGVAGNNTVALGTDLSFDTATGNFTKCNAGLSFSSSDLIASLALNDKGDTVSASYY
O.sativa             VHDLGTPGLKGILSIPFP---YQKSAKAEVQYLHPHAGLNAIVGLN-ANPLVSFSGVFGTGAFAVGTDVAFDTATGDFTKYNAGLSHTTPDLTAALLLNNKGDSLAASYY
S.cerviseae          FS-KIAPGWKGDVNAFLTPQ-SIKNAKFNLSYAQKSFAARTSIDIL-QPKDFVGSVTLGHRGFVGGTDIAYDTAAGLCARYAMSIGYLAREYSFILSTNN-RQCATASFF
Clustal Consensus    .      . *   .  .       :..* :  * :          :      .  : . *      * :  :*::    ::   .:      :        :     .*

                          230       240       250       260       270       280       290       300       310
                     ....|....|....|....|....|....|....|....|....|....|....|....|....|....|....|....|....|....|
H.sapiens            QKVCEDLDTSV--NLAWTSGTNCTRFGIAAKYQLDPTASISAKVNNSSLIGVGYTQTLRPGVKLTLSALVDGKSINAGGHKVGLALELEA
C.griseus            QKVCEDFDTSV--NLAWTSGTNCTRFGIAAKYQLDPTASISAKVNNSSLIGVGYTQTLRPGVKLTLSALVDGKSFNAGGHKLGLALELEA
B.taurus             QKVCEDLDTSV--NLAWTSGTNCTRFGIAAKYQLDPTASISAKVNNSSLIGVGYTQTLRPGVKLTLSALVDGKSINAGGHKLGLPLELEA
S.scrofa             QKVCEDLDTSV--NLAWTSGTNCTRFGIAAKYQLDPTASISAKVNNSSLIGVGYTQTLRPGVKLTLSALVDGKSINAGGHKLGLALELEA
M.mulatta            QKVCEDLDTSV--NLAWTSGTNCTRFGIAAKYQLDPTASISAKVNNSSLIGVGYTQTLRPGVKLTLSALVDGKSINAGGHKLGLALELEA
O.cuniculus          QKVCEDLDTSV--NLAWTSGTNCTRFGIAAKYQLDPTASISAKVNNSSLIGVGYTQTLRPGVKLTLSALVDGKSINAGGHKLGLALELEA
M.musculus           QKVCEDFDTSV--NLAWTSGTNCTRFGIAAKYQLDPTASISAKVNNSSLIGVGYAQTLRPGVKLTLSALVDGKSFNAGGHKLGLALELEA
R.norvegicus         QKVCEDFDTSV--NLAWTSGTNCTRFGIAAKYQLDPTASISAKVNNSSLIGVGYTQTLRPGVKLTLSALVDGKSFNAGGHKLGLALELEA
B.mutus              QKVCEDLDTSV--NLAWTSGTNCTRFGIAAKYQLDPTASISAKVNNSSLIGVGYTQTLRPGVKLTLSALVDGKSINAGGHKLGLALELEA
M.brandtii           QKVCEDLDTSV--NLAWTSGTNCTRFGIAAKYQLDPTASISAKVNNSSLIGVGYTQTLRPGVKLTLSALVDGKSINAGGHKLGLALELEA
X.laevis             QKVSDKMETAV--NLAWTSGNNSTRFGIAAKYQLDSHAAISAKVNNSSLVGVGYTQTLRPGVKLTLSALVDGKNINAGGHKLGLGLELEA
C.mydas              QKVSDNLDTAV--NLAWTAGSNSTRFGIAAKYQLDSTASISAKVNNSSLVGVGYTQTLRPGVKLTLSALVDGKSINSGGHKLGLGLELEA
A.platyrhynchos*     QKVSDNLETAV--NLSWTTGSNSTRFGIAAKYKLDSTASISAKVNNSSLVGVGYTQTLRPGVKLTLSALIDGKSINAGGHKLGLGLELEA
C.brachyrhynchos*    QKVSDSLETAV--NLAWTAGSNSTRFGIAAKYKLDSTASISAKVNNSSLVGVGYTQTLRPGVKLTLSALIDGKSINAGGHKLGLGLELEA
C.anna*              QKVSDSLETAV--NLAWTAGSNSTRFGIAAKYKLDSTASISAKVNNSSLVGVGYTQTLRPGVKLTLSALIDGKSINAGGHKLGLGLELEA
B.rhinoceros*        QKVSDNLETAV--NLAWTAGSNSTRFGIAAKYKLDSTASISAKVNNSSLVGVGYTQTLRPGVKLTLSALIDGKSINAGGHKLGLGLELEA
A.forsteri*          QKVSDNLETAV--NLAWTAGSNSTRFGIAAKYKLDSTASISAKVNNSSLVGVGYTQTLRPGVKLTLSALIDGKSINAGGHKLGLGLELEA
S.camelus*           QKVSDNLETAV--NLAWTAGSNSTRFGIAAKYKLDSTASISAKVNNSSLVGVGYTQTLRPGVKLTLSALIDGKSINAGGHKLGLGLELEA
M.gallopavo          QKVSDNLETAV--NLAWTAGSNSTRFGIAAKYKLDSTASISAKVNNSSLVGVGYTQTLRPGVKLTLSALIDGKSINAGGHKLGLGLELEA
G.gallus             QKVSDNLETAV--NLAWTAGSNSTRFGIAAKYKLDSTASISAKVNNSSLVGVGYTQTLRPGVKLTLSALIDGKSINAGGHKLGLGLELEA
D.rerio              QKVSDKLETAV--NLAWTAGSNSTRFGIAAKYQLDKDASISAKVNNTSLVGVGYTQSLRPGIKLTLSALVDGKSINSGGHKLGLGLELEA
S.salar              QKVNDKLETAV--NLAWTAGSNSTRFGIAAKYQLDSSASISAKVNNASLVGVGYTQTLRPGMKLVLSALVDGKSINAGGHKLGLGLELEA
A.thaliana           HHFDEFKRTAAVGEVYRKFSTNENTITVGGLYAIDHSTAVKAKLNNHGTLGALLQHEVLPRSLVTVSSEIDTKALEKH-PRFGLSLALKP
S.tuberosum          HTVKPVTNTAVGAELTHSFSSNENTLTIGTQHLLDPLTTVKARVNSYGKASALIQHEWRPKSLFTISGEVDTRAIEKS-AKIGLAVALKP
O.sativa             HKVSK--TSAVGAELAHSFSSNENTLTFGTQHALDELTTVKARFNNFGMASALIQHEFRPKSLVTISTEVDTKAIDKS-SKVGLSLVLKP
S.cerviseae          QNVNRYLQVGTKATLQSKTSSNM-NIEFVTRYVPDSISQVKAKIADSGLTTLSYKRNLNKDISLGVGMSFNALQLTEPVHKFGWSLSFSP
Clustal Consensus    : .       ..   :  . ..*   : .    :  *  : :.*:. . .        :        . :.  .:   :     :.*  : :.
```

SUPPLEMENTAL FIGURE 1. Evolutionary conservation of NTE and cysteines in various VDAC-2 proteins. Multiple sequence alignment (MSA) of VDAC-2 sequence from various organisms, generated using Clustal Omega (1,2). Cysteines are highlighted in yellow. Note how those VDAC-2 sequences with an abundance of cysteines also possess additional residues at the N-terminal region (this corresponds to the N-terminal extension (NTE), which we describe in this study). The length of this NTE region varies across various species. Also notable is the existence of sequence variation in the NTE, although all cysteines are highly conserved. NTE could therefore have been acquired by the Cys-containing VDAC-2 barrel, in the course of evolution. Organisms for which only partial sequence was available have been marked with *****.

```
                                10        20        30        40        50        60        70        80        90       100       110
                       ....|....|....|....|....|....|....|....|....|....|....|....|....|....|....|....|....|....|....|....|....|....|
H.sapiens              -------------MAVPPTYADLGKSARDVFTKGYGF-GLIKLDLKTKSENGLEFTSSGSANTETTKVTGSLETKYRWTEYGLTFTEKWNTDNTLGTEITVEDQLARGLK
C.griseus              -------------MAVPPTYDYLGKPARDVFTKGYGF-GLIKLDLKTKSENGLEFTSSGSANTETTKVNGSLETKYRRTQYELTFTEKWNTDNTLV--------------
B.taurus               -------------MAVPPTYADLGKSARDVFTKGYGF-GLIKLDLKTKSENGLEFTSSGSANTETTKVTGSLETKYRWTEYGLTFTEKWNTDNTLGTEITVEDQLARGLK
S.scrofa               -------------MAVPPTYADLGKSARDVFTKGYGF-GLIKLDLKTKSENGLEFTSSGSANTETTKVTGSLETKYRWTEYGLTFTEKWNTDNTLGTEITVEDQLARGLK
M.mulatta              -------------MAVPPTYADLGKSARDVFTKGYGF-GLIKLDLKTKSENGLEFTSSGSANTETTKVTGSLETKYRWTEYGLTFTEKWNTDNTLGTEITVEDQLAHGLK
O.cuniculus            -------------MAVPPTYADLGKSARDVFTKGYGF-GLIKLDLKTKSENGLEFTSSGSANTETTKVTGSLETKYRWTEYGLTFTEKWNTDNTLGTEITVEDQLARGLK
M.musculus             MCSFFLVLLLWQNMAVPPTYADLGKSARDVFTKGYGF-GLIKLDLKTKSENGLEFTSSGSANTETTKVNGSLETKYRWTEYGLTFTEKWNTDNTLGTEITVEDQLARGLK
R.norvegicus           -------------MAVPPTYADLGKSARDVFTKGYGF-GLIKLDLKTKSENGLEFTSSGSANTETTKVNGSLETKYRWTEYGLTFTEKWNTDNTLGTEITVEDQLARGLK
B.mutus                -----------QKMAVPPTYADLGKSARDVFTKGYGF-GLIKLDLKTKSENGLEFTSSGSANTETTKVTGSLETKYRWTEYGLTFTEKWNTDNTLGTEITVEDQLARGLK
M.brandtii             -------------MAVPPTYIDLGKSARDVFTKGYGF-GLIKLELKTKSESGLEFTSSGSANTETTKVAGSLETKYRWTEYGLTFTEKWNTDNTLGTEITVEDQLARGLK
X.laevis               -------------MAIPPAYVDLGKSARDIFTKGYGF-GFIKLDLKTKSENGLEFTSSGSANAETSKVSGNLETKYKWAEYGLTFTEKWNTDNTLGTEITVEDQLAKGLK
C.mydas                -------------MAVPPAYADLGKSARDVFTKGYGF-GLIKLDLKTKSENGLEFTSSGSANSETSKVTGSLETKYKWGEYGLTFTEKWNTDNTLGTEITLEDQLAHGLK
A.platyrhynchos*       -----------------------------------------------------EFTSSGSANSETSKVSGSLETKYRWVEYGLMFTEKWNTDNTLGTEITLEDQLARGLK
C.brachyrhynchos*      -----------QKMAVPPAYADLGKSARDIFTKGYGF-GLIKLDLKTRSENGLEFTSSGSANSETNKVSGSLETKYKWVEYGLMFTEKWNTDNTLGTEITLEDQLARGLK
C.anna*                ------------KMAVPPAYADLGKSARDIFTKGYGF-GLIKLDLKTRSENGLEFTSSGSANSETSKVSGSLETKYKWVEYGLMFTEKWNTDNTLGTEITLEDKLARGLK
B.rhinoceros*          -----------------------------------------------------EFTSSGSANSETNKVSGSLETKYRWVEYGLMFTEKWNTDNTLGTEITLEDQLARGLK
A.forsteri*            -----------QKMAVPPAYADLGKSARDVFTKGYGF-GLIKLDLKTKSENGLEFTSSGSANSETSKVSGSLETKYKWVEYGLMFTEKWNTDNTLGTEITLEDQLARGLK
S.camelus*             -----------QKMAVPPAYADLGKSARDVFTKGYGF-GLIKLDLKTKSENGLEFTSSGSANSETSKVSGSLETKYKWVEYGLMFTEKWNTDNTLGTEITLEDQLAQGLK
M.gallopavo            PC----FFSPPQKMAVPPAYADLGKSARDVFTKGYGF-GLIKLDLKTKSENGLEFTSSGSANSETSKVSGSLETKYRWVEYGLMFTEKWNTDNTLGTEITLEDQLARGLK
G.gallus               -------------MAVPPAYADLGKSARDVFTKGYGF-GLIKLDLKTKSENGLEFTSSGSANSETSKVSGSLETKYRWVEYGLMFTEKWNTDNTLGTEITLEDQLARGLK
D.rerio                -------------MAVPPTYVDLGKSARDIFTKGYGF-GLIKLDLKTRSENGLEFKSSGSANTETSKVAGTLETKYKWAEHGLTFTEKWNTDNTLGTEITLEDQLTKGLK
S.salar                -------------MAVPPTYVDLGKSAKDVFTKGYGF-GLIKLDLKTKSENGLEFTSTGSANTETSKVAGTLETKYKWAEHGLTFTEKWNTDNTLGTEITLEDQLAKGLQ
A.thaliana             ------------MVKGPGLYTEIGKKARDLLYKDHNS--DQKFSITTFSPAGVAITSTGTKKGD--LLLGDVAFQSR--RKNITTDLKVCTDSTFLITATVDE-AAPGLR
S.tuberosum            ------------MGKGPGLYTEIGKKARDLLYKDYQS--DHKFSITTYSPTGVVITSSGSKKGD--LFLADVNTQLK--NKNVTTDIKVDTNSNLFTTITVDE-AAPGLK
O.sativa               -------------MVGPGLYPEIGKKARDLLYRDYQT--DHKFTLTTYTSNGVAITATSTKKAD--LIFGEIQSQIK--NKNITVDVKANSDSNVVTTVTVDE-LTPGLK
S.cerviseae            --------------MSPPVYSDISRNINDLLNKDFYHATPAAFDVQTTTANGIKFSLKAKQPVKDGPLSTNVEAKLNDKQTGLGLTQGWSNTNNLQTKLEFAN-LTPGLK
Clustal Consensus                                                            :. ...   .   .   :  :  . .  :       .  ...

                               120       130       140       150       160       170       180       190       200       210       220
                       ....|....|....|....|....|....|....|....|....|....|....|....|....|....|....|....|....|....|....|....|....|....|
H.sapiens              LTFDSSFSPNTGKKNAKIKTGYKREHINLGCDMDFDIAGPSIRGALVLGYEGWLAGYQMNFETAKSRVTQSNFAVGYKTDEFQLHTNVND-GTEFGGSIYQKVNKKLETA
C.griseus              -----------GGGGAKIKTGYKREHINLGCDVDFDIAGPSIWGALVFGYEGWLAGYQMNFETSKSRVTQSNFAVVYRTNEFQLHTNVIR-GTEFGGSIYQKVNKKLETA
B.taurus               LTFDSSFSPNTGKKNAKIKTGYKREHINLGCDVDFDIAGPSIRGALVLGYEGWLAGYQMNFETAKSRVTQSNFAVGYKTDEFQLHTNVND-GTEFGGSIYQKVNKKLETA
S.scrofa               LTFDSSFSPNTGKKNAKIKTGYKREHVNLGCDVDFDIAGPSIRGALVLGYEGWLAGYQMNFETAKSRVTQSNFAVGYKTDEFQLHTNVND-GTEFGGSIYQKVNKKLETA
M.mulatta              LTFDSSFSPNTGKKNAKIKTGYKREHINLGCDVDFDIAGPSIRGALVLGYEGWLAGYQMNFETAKSRVTQSNFAVGYKTDEFQLHTNVND-GTEFGGSIYQKVNKKLETA
O.cuniculus            LTFDSSFSPNTGKKNAKIKTGYKREHINLGCDVDFDIAGPSIRGALVLGYEGWLAGYQMNFETAKSRVTQSNFAVGYKTDEFQLHTNVND-GTEFGGSIYQKVNKKLETA
M.musculus             LTFDSSFSPNTGKKNAKIKTGYKREHINLGCDVDFDIAGPSIRGALVLGYEGWLAGYQMNFETSKSRVTQSNFAVGYKTDEFQLHTNVND-GTEFGGSIYQKVNKKLETA
R.norvegicus           LTFDSSFSPNTGKKNAKIKTGYKREHINLGCDVDFDIAGPSIRGALVLGYEGWLAGYQMNFETSKSRVTQSNFAVGYKTDEFQLHTNVND-GTEFGGSIYQKVNKKLETA
B.mutus                LTFDSSFSPNTGKKNAKIKTGYKREHINLGCDVDFDIAGPSIRGALVLGYEGWLAGYQMNFETAKSRVTQSNFAVGYKTDEFQLHTNVND-GTEFGGSIYQKVNKKLETA
M.brandtii             LTFDSSFSPNTGKKNAKIKTGYKREHINLGCDVDFDMAGPSIRGALVLGYEGWLAGYQMNFETAKSRVTQSNFAVGYKTDEFQLHTNVND-GTEFGGSIYQKVNKKLETA
X.laevis               LTFDSSFSPNTGKKNAKVKSAYKREHLNVGCDMDFDIAGPSVRGAVVFGYEGWLAGYQMTFESSKSRVSQSNFAVGYKTDEFQLHTNVND-GTDFGGSVYQKVNDKLETA
C.mydas                LTFDSSFSPNTGKKNAKIKTGYKREHINLGCDMDFDIAGPSIRGAFVFGYEGWLAGYQMTFETTKSRVTQSNFAVGYKTDEFQLHTNVND-GTEFGGSIYQKVNDKLETA
A.platyrhynchos*       LTFDSSFSPNTGKKSAKVKTGYKREHINMGCDMDFDIAGPSIRGALVVGYEGWLAGYQMTFETAKSRVTQSNFAVGYKTDEFQLHTNVND-GTEFGGSIYQKVNDKLETA
C.brachyrhynchos*      LTFDSTFSPNTGKKSAKIKSGYKREHINIGCDMDFDIAGPSIRGALVLGYEGWLAGYQMTFETAKSRVTQSNFAVGYKTDEFQLHTNVND-GTEFGGSIYQKVNDKLETA
C.anna*                LTFDSSFSPNTGKKSAKVKSGYKREHFNMGCDMDFDIAGPSIRGALVVGYEGWLAGYQMTFETAKNRVTQSNFAVGYKTDEFQLHTNVND-GTEFGGSIYQKVNDKLETA
B.rhinoceros*          LTFDSTFSPNTGKKSAKVKTGYKREHINLGCDMDFDIAGPSIRGALVVGYEGWLAGYQMIFETAKSRVTQSNFAVGYKTDEFQLHTNVND-GTEFGGSIYQKVNDKLETA
A.forsteri*            LTFDSSFSPNTGKKSAKVKTGYKREHINIGCDMDFDIAGPSIRGALVVGYEGWLAGYQMTYETTKSRVTQSNFAVGYKTDEFQLHTNVND-GTEFGGSIYQKVNDKLETA
S.camelus*             LTFDSSFSPNTGKKSAKVKTGYKREHINIGCDMDFDIAGPSIRGALVVGYEGWLAGYQMTFETAKSRVTQSNFAVGYKTDEFQLHTNVND-GTEFGGSIYQKVNDKLETA
```

```
M.gallopavo        LTFDSSFSPNTGKKSAKIKTGYKREHINMGCDMDFDIAGPSIRGALVLGYEGWLAGYQMTFETAKSRVTQSNFAVGYKTDEFQLHTNVNRDLTGFGGSIYQKVNDKLETA
G.gallus           LTFDSSFSPNTGKKSAKIKTGYKREHINMGCDMDFDIAGPSIRGALVLGYEGWLAGYQMTFETAKSRVTQSNFAVGYKTDEFQLHTNVND-GTEFGGSIYQKVNEKLETA
D.rerio            LTFDSSFSPNTGKKSGKIKSSYQREHINLGCDVDYDINGTAVHGALVLGLDGWLAGYQMTFEAGRNRITQSNFAVGYKTDEFQLHTNVND-GTEFGGSIYQKVNDNLETA
S.salar            FTFDSSFSPNTGKKSGKIKTGYKREHINLGCDVDYDINGTAVHGVAVVGYEGWLAGYQMTFEAGKNRVTQSNFAVGYKTDEFQLHTNVND-GTEFGGSIYQKVNDQLETA
A.thaliana         SIFSFKVP---DQNSGKVELQYLHEYAGISTSMGL-TQNPTVNFSGVIGSNVLAVGTDVSFDTKSGNFTKINAGLSFTKEDLIASLTVNDKGDLLNASYYHIVNPLFNTA
S.tuberosum        TILSFRVP---DQRSGKLEVQYLHDYAGICTSVGL-TANPIVNFSGVVGTNIIALGTDVSFDTKTGDFTKCNAGLSFTNADLVASLNLNNKGDNLTASYYHTVSPLTSTA
O.sativa           SILSFAVP---DQRSGKFELQYSHDYAGVSASIGL-TASPVVNLSSVFGTKALAVGADVSLDTATGNLTKYNAGLSFSNDDLIASLNLNNKGDSLTASYYHIVNH-SATA
S.cerviseae        NELITSLTPGVAK-SAVLNTTFTQPFFTARGAFDLCLKSPTFVGDLTMAHEGIVGGAEFGYDISAGSISRYAMALSYFAKDYSLGATLNNEQI-TTVDFFQNVNAFLQVG
Clustal Consensus                 . .:  : : .      .        .    ... .    * :.  :     .::   .: :   :     .:         . :: *.    ..

                           230       240       250       260       270       280       290       300
                   ....|....|....|....|....|....|....|....|....|....|....|....|....|....|....|....|.
H.sapiens          --VNLAWTAGNSNTRFGIAAKYQIDPDACFSAKVNNSSLIGLGYTQTLKPGI-KLTLSALLDGKNVNAGGHKLGLGLEFQA
C.griseus          --VNLAWTAGNSNICFGIATKYQVDPDAGFLDKVNNSSRISLEYAKARYQTDSQLCWTARTS---MR-----VATSLV---
B.taurus           --VNLAWTAGNSNTRFGIAAKYQIDPDACFSAKVNNSSLIGLGYTQTLKPGI-KLTLSALLDGKNVNAGGHKLGLGLEFQA
S.scrofa           --VNLAWTAGNSNTRFGIAAKYQIDPDACFSAKVNNSSLIGLGYTQTLKPGI-KLTLSALLDGKNVNAGGHKLGLGLEFQA
M.mulatta          --VNLAWTAGNSNTRFGIAAKYQIDPDACFSAKVNNSSLIGLGYTQTLKPGI-KLTLSALLDGKNVNAGGHKLGLGLEFQA
O.cuniculus        --VNLAWTAGNSNTRFGIAAKYQIDPDACFSAKVNNSSLIGLGYTQTLKPGI-KLTLSALLDGKNVNAGGHKLGLGLEFQA
M.musculus         --VNLAWTAGNSNTRFGIAAKYQVDPDACFSAKVNNSSLIGLGYTQTLKPGI-KLTLSALLDGKNVNAGGHKLGLGLEFQA
R.norvegicus       --VNLAWTAGNSNTRFGIAAKYQVDPDACFSAKVNNSSLIGLGYTQTLKPGI-KLTLSALLDGKNVNAGGHKLGLGLEFQA
B.mutus            --VNLAWTAGNSNTRFGIAAKYQIDPDACFSAKVNNSSLIGLGYTQTLKPGI-KLTLSALLDGKNVNAGGHKLGLGLEFQA
M.brandtii         --VNLAWTAGNSNTRFGIAAKYQIDPEACFSAKVNNSSLIGLGYTQTLKPGI-KLTLSALLDGKNVNAGGHKLGLGLEFQA
X.laevis           --INLAWTAGNSNTRFGIAAKYQIDSDASFSAKVNNSSLIGLGYTQTLKPGI-KLTLSTLVDGKNINAGGHKLGLGLEFEA
C.mydas            --VNLAWTAGNSNTRFGIAAKYQIDPDASFSAKVNNSSLIGLGYTQTLKPGI-KLTLSALLDGKNVNAGGHKLGLGLEFEA
A.platyrhynchos*   --VNLAWTAGNSNTRFGIAAKYQIDPDASFSAKVNNSSLIGLGYTQTLKPGI-KLTLSALLDGKNVNAGGHKLGLGLEFEA
C.brachyrhynchos*  --VNLAWTAGNSNTRFGIAAKYQIDPDASFSAKVNNSSLIGLGYTQTLKPGI-KLTLSALLDGKNVNAGGHKLGLGLEFEA
C.anna*            --VNLAWTAGNSNTRFGIAAKYQIDPDASFSAKVNNSSLIGLGYTQTLKPGI-KLTLSALLDGKNVNAGGHKLGLGLEFEA
B.rhinoceros*      --VNLAWTAGNSNTRFGIAAKYQIDPDASFSAKVNNSSLIGLGYTQTLKPGI-KLTLSALLDGKNVNTGGHKLGLGLEFEA
A.forsteri*        --VNLAWTAGNSNTRFGIAAKYQIDPDASFSAKVNNSSLIGLGYTQTLKPGI-KLTLSALLDGKNVNAGGHKLGLGLEFEA
S.camelus*         --VNLAWTAGNSNTRFGIAAKYQIDPDASFSAKVNNSSLIGLGYTQTLKPGI-KLTLSALLDGKNVNAGGHKLGLGLEFEA
M.gallopavo        --VNLAWTAGNSNTRFGIAAKYQIDPDASFSAKVNNSSLIGLGYTQTLKPGI-KLTLSALLDGKNVNAGGHKLGLGLEFEA
G.gallus           --VNLAWTAGNSNTRFGIAAKYQIDPDASFSAKVNNSSLIGLGYTQTLKPGI-KLTLSALLDGKNVNAGGHKLGLGLEFEA
D.rerio            --VNLAWTAGNSNTRFGIAAKYQIDSDAAFSAKVNNSSLVGLGYTQTLKPGI-KLTLSALLDGKNINAGGHKMGLGLEFEA
S.salar            --VNLAWTAGNSNTRFGIAAKYQIDADAAFSAKVNNSSLVGLGYTQTLKPGI-KLTLSALLDGKNINAGGHKLGLGLEFEA
A.thaliana         VGAEVSHKLSSKDSTITVGTQHSLDPLTSVKARVNSAGIASALIQHEWKPKS-FFTISGEVDTKSIDK-SAKVGLALALKP
S.tuberosum        VGAEVNHSFSTNENIITVGTQHRLDPLTSVKARINNFGKASALLQHEWRPKS-LFTVSGEVDTKSVDK-GAKFGLALALKP
O.sativa           VGAELTHSFSSNENSLTFGTQHTLDPLTVVKARFNNSGKASALLQHEWRPKS-VWTISAEVDTKAIDK-SSKVGIAVALKP
S.cerviseae        AKATMNCKLPNSNVNIEFATRYLPDASSQVKAKVSDSGIVTLAYKQLLRPGV-TLGVGSSFDALKLSEPVHKLGWSLSFDA
Clustal Consensus      :  .  ..:  : ..:::  *  : .  :... .       :               .   :      .. .:
```

SUPPLEMENTAL FIGURE 2. Evolutionary conservation of cysteines in various VDAC-1 proteins. MSA of VDAC-1 sequence from various organisms generated using Clustal Omega (1,2). Cysteines are highlighted in yellow. The first conserved cysteine (C127 in hVDAC-1) is retained in most species, except in a few plants and yeast. The second conserved cysteine (C232 in hVDAC-1) is restricted to higher mammals, with the notable exception of *C. griseus* (Chinese hamster), which also shows an overall lower sequence similarity to other mammalian VDAC-1 sequences. Organisms for which only partial sequence was available have been marked with *****.

```
                                  10        20        30        40        50        60        70        80        90       100       110
                         ....|....|....|....|....|....|....|....|....|....|....|....|....|....|....|....|....|....|....|....|....|....|
H.sapiens                -------------MCNTPTYCDLGKAAKDVFNKGYGFGMVKIDLKTKSCSGVEFSTSGHAYTDTGKASGNLETKYKVCNYGLTFTQKWNTDNTLGTEISWENKLAEGLKL
C.griseus                -------------MCNTPTYCDLGKAAKDVFNKGYGFGMVKIDLRTKSCSGVEFSTSGHAYTDTGKASGNLETKYKVCDYGLTFTQKWNTDNTLGTEVSWENKLADGLKL
B.taurus                 -------------MCNTPTYCDLGKAAKDVFNKGYGFGMVKIDLRTKSCSGVEFSTSGHAYTDTGKASGNLETKYKICNYGLTFTQKWNTDNTLGTEISWENKLAEGLKL
S.scrofa                 -------------MCNTPTYCDLGKAAKDVFNKGYGFGMVKIDLRTKSCSGVEFSTSGHAYTDTGKASGNLETKYKICDHGLTFTQKWNTDNTLGTEISLENKLAEGLKL
M.mulatta                -------------MCNTPTYCDLGKAAKDVFNKGYGFGMVKIDLKTKSCSGVEFSTSGHAYTDTGKASGNLETKYKVCNYGLTFTQKWNTDNTLGTEISWENKLAEGLKL
O.cuniculus              -------------MCNTPTYCDLGKAAKDVFNKGYGFGMVKIDLRTKSCSGVEFSTSGHAYTDTGKASGNLETKYKVCNYGLTFTQKWNTDNTLGTEISLENKLAEGLKL
M.musculus               -------------MCNTPTYCDLGKAAKDVFNKGYGFGMVKIDLKTKSCSGVEFSTSGHAYTDTGKASGNLETKYKVCNYGLTFTQKWNTDNTLGTEISWENKLAEGLKL
R.norvegicus             -------------MCSTPTYCDLGKAAKDVFNKGYGFGMVKIDLKTKSCSGVEFSTSGHAYTDTGKASGNLETKYKVCNYGLIFTQKWNTDNTLGTEISWENKLAEGLKL
B.mutus                  -------------MCNTPTYCDLGKAAKDVFNKGYGFGMVKIDLRTKSCSGVEFSTSGHAYTDTGKASGNLETKYKICNYGLTFTQKWNTDNTLGTEISWENKLAEGLKL
M.brandtii               --------MLKSQMCNTPTYCDLGKAAKDVFNKGYGFGMVKIDLRTKSCSGVEFSTSGHAYTDTGKASGNLETKYKICNYGLTFTQKWNTDNTLGTEISWENKLAEGLKL
X.laevis                 -------------MAVPPTYADLGKSARDVFNKGYGFGLVKLEVKTKSSSGVEFTSSGSSNTDTGKASGNLETKYKMKETGLTFTQKWNTDNTLGTEVALEDKLAKGLKL
C.mydas                  --------------------------------------MIKLELKTKSSSGVEFTTTGSSNTDTGKASGNLETKYKIKDYGLTFTQKWNTDNTLGTEVSMEDKLAEGLKL
A.platyrhynchos*         FAYYWNGDKCSYPMAVPPSYSDLGKSARDVFNKGYGFGMVKLELKTKSSSGVEFTATGSSNTDTGKASGSLETKYKIKDYGLTFLQKWNTDNTLGTEVSMEDQLAEGLKV
C.brachyrhynchos*        -----------------------------------GFGMVKLELKTKSSSGVEFTATGSSNTDTGKASGSLETKYKVKDYGLTFTQKWNTDNTLGTEVSVEDQLAEGLKV
C.anna*                  ------------PMAVPPSYSDLGKSARDVFNKGYGFGMVKLELKTKSSTGVEFTATGSSNTDTGKASGSLETKYKVKDYGLTFTQKWNTDNTLGTEVSVEDQLAEGLKV
B.rhinoceros*            ------------PMAVPPTYSDLGKSARDVFNKGYGFGMVKLELKTKSSSGVEFTATGSSNTDTGKASGSLETKYKIKDYGLTFTQKWNTDNTLGTEVAMEDQLAEGLKV
A.forsteri*              ------------PMAVPPSYNDLGKCARDVFNKGYGFGMVKLELKTKSSSGVEFTATGSSNTDTGKASGSLETKYKIKDYGLTFTQKWNTDNTLGTEVSMEDQLAEGLKV
S.camelus*               -----------------------------------GFGMVKLELKTKSSTGVEFTATGSSNTDTGKASGSLETKYKIKDCGLTFTQKWNTDNTLGTEVSVEDQLAEGLKV
M.gallopavo              -------------MAVPPSYSDLGKAARDVFNKGYGFGMVKLELKTKSSSGVEFTATGSSNTDTGKALGNLETKYKIKDYGLTFIQKWNTDNTLGTEVSMEDQLAEGLKV
G.gallus*                ------------PMAVPPSYSDLGKAARDVFNKGYGFGMVKLELKTKSSSGVEFTATGSSNTDTGKALGNLETKYKIKDYGLTFIQKWNTDNTLGTEVSMEDQLAEGLKV
D.rerio                  -------------MAVPPAYADLGKSAKDIFSKGYGFGTVKLDLKTKSQSGVEFTTGGSSNTDTGKAAGNLETKYKVKELGLSLNQKWNTDNVLTTEVTLEDQLAKGLKL
S.salar                  -------------MAVPPAYSDLGKAAKDIFGKGYGFGIVKLDLKTKAQSGVEFATSGSSNTDTGKAAGNLETKYKVKELGLSFTQKWNTDNTLTTEVSMEDQLAKGLKL
A.thaliana               ------------MVKGPGLYTEIGKKARDLLYRDYQG-DQKFSVTTYSSTGVAITTTGTNKGS--LFLGDVAT--QVKNNNFTADVKVSTDSSLLTTLTFDE-PAPGLKV
O.sativa                 --------------MAPGLYTDIGKKTRDLLYRDYGT-HHKFTLTTCTPEGVTITAAGTRKNE--SVFGELQT--QLKNKKLTVDVKANSESDLLTTVTVDEFGTPGLKS
Clustal Consensus                                                 *: : * :  ** ::: *    .     *.: *  :: :  :    * .::. * * :: ::  : ***

                                 120       130       140       150       160       170       180       190       200       210       220
                         ....|....|....|....|....|....|....|....|....|....|....|....|....|....|....|....|....|....|....|....|....|....|
H.sapiens                TLDTIFVPNTGKKSGKLKASYKRDCFSVGSNVDIDFSGPTIYGWAVLAFEGWLAGYQMSFDTAKSKLSQNNFALGYKAADFQLHTHVND-GTEFGGSIYQKVNEKIETSI
C.griseus                TLDTIFVPNTGKKSGKLKASFRRDCFSLGSNVDIDFSGPTIYGWAVLAFEGWLAGYQMSFDTAKSKLSQNNFALGYKAEDFQLHTHVND-GTEFGGSIYQKVNEKVETSV
B.taurus                 TLDTIFVPNTGKKSGKLKASYKRDCFSLGSNVDIDFSGPTIYGWAVLAFEGWLAGYQMSFDTAKSKLSQNNFALGYKAADFQLHTHVND-GTEFGGSIYQKVNEKIETSI
S.scrofa                 TLDTIFVPNTGKKSGKLKASYKRECFSIGSNVDIDFAGPTIYGWAVLALEGWLAGYQMSFDTAKSKLSQNNFALGYKAADFQLHTHVND-GTEFGGSIYQKVNEKIETSI
M.mulatta                TLDTIFVPNTGKKSGKLKASYKRDCFSVGSNVDIDFSGPTIYGWAVLAFEGWLAGYQMSFDTAKSKLSQNNFALGYKAADFQLHTHVND-GTEFGGSIYQKVNEKIETSI
O.cuniculus              TLDTIFVPNTGKKSGKLKASYKRDCFSLGSNVDIDFSGPTIYGWAVLAFEGWLAGYQMSFDTAKSKLSQNNFALGYKAADFQLHTHVND-GTEFGGSIYQKVNEKIETSI
M.musculus               TLDTIFVPNTGKKSGKLKASYRRDCFSLGSNVDIDFSGPTIYGWAVLAFEGWLAGYQMSFDTAKSKLSQNNFALGYKAADFQLHTHVND-GTEFGGSIYQKVNERIETSI
R.norvegicus             TVDTIFVPNTGKKSGKLKASYRRDCFSVGSKVDIDFSGPTIYGWAVLAFEGWLAGYQMSFDTAKSKLCQNNFALGYKAEDFQLHTHVND-GTEFGGSIYQRVNEKIETSI
B.mutus                  TLDTIFVPNTGKKSGKLKASYKRDCFSLGSNVDIDFSGPTIYGWAVLAFEGWLAGYQMSFDTAKSKLSQNNFALGYKAADFQLHTHVND-GTEFGGSIYQKVNEKIETSI
M.brandtii               TLDTTFVPNTGKKSGKLKASYKRDCFSLGSNVDIDFSGPTIYGWAVLGFEGWLAGYQMSFDTAKSKLSQNNFALGYKAADFQLHTHVND-GTEFGGSIYQKVNEKTETSI
X.laevis                 SLDTTFVPNTGKKSAKLKTSYKRDYANLGIDIDLDLAGPTIYGWGVLGYQGWLAGYQMAFDTAKSRLAQNNFALGYTAGDFQLHTNVND-GTEFGGSVYQKVSKDVETSV
C.mydas                  TLDTIFVPNTGKKSGKLKTSYKREYVNLGCNIDVDLSGPTIHGWAVLGYEGWLAGYQMAFDTAKSKLSQNNFALGYKAGDFQLHTNVND-GTEFGGSIYQKVNDKVETSV
A.platyrhynchos*         ALDTVFVPNTGKKSGKLKTSYKREYLNLGCNIDIDLSGPTIYGWAVLGYEGWLAGYQMAFDTAKSKLSQNNFALGYKAGDFQLHTNVND-GTEFGGSIYQKVNNKVETSV
C.brachyrhynchos*        ALDSTFVPNTGKKSGKLKTSYKRDYVHLGCNIDIDLSGPTIYGWAVLGYEGWLAGYQMAFDTAKSKLSQNNFALGYKAGDFQLHTNVND-GTEFGGSIYQKVNNKVETSV
C.anna*                  ALDTIFVPNTGKKSGKLKTSYKRDYVNLGCNIDIDLSGPTIYGWAVLGYEGWLAGYQMAFDTAKSKLSQNNFALGYKAGDFQLHTNVND-GTEFGGSIYQKVNNKVETSV
B.rhinoceros*            ALDTTFVPNTGKKSGKLKTSYKRDHLNIGCNVDIDLSGPTIYGWAVLGYEGWLAGYQMAFDTAKYKLSQNNFSLGYKAGDFQLHTNVND-GTEFGGSIYQKVNDKVETSV
A.forsteri*              ALDTTFVPNTGKKSGKLKTSYKREHVNLGCNIDIDLSGPTVYGWAVLGYEGWLAGYQMAFDTAKSKLSQNNFALGYKAGDFQLHTNVND-GTEFGGSIYQKVNNKVETSV
S.camelus*               ALDTVFVPNTGKKSGKLKTSYKREYVNLGCNIDIDLSGPTIYGWAVLGYEGWLAGYQMAFDTAKSKLSQNNFALGYKAGDFQLHTNVND-GTEFGGSIYQKVNNKVETSV
M.gallopavo              ALDTIFVPNTGKKSGKLKTSYKREYVNLGCNIDIDLSGPTIYGWAVLGYEGWLAGYQMAFDTAKSKLSQNNFALGYKAGDFQLHTNVND-GTEFGGSIYQKVNNKVETSV
G.gallus*                ALDTIFVPNTGKKSGKLKTSYRRDYVNLGCNIDIDLSGPTIYGWAVLGYEGWLAGYQMAFDTAKSKLSQNNFALGYKAGDFQLHTNVND-GTEFGGSIYQKVNNKVETSV
```

```
D.rerio             GLDTSFVPNTGKKSAKLKTGYKREYMNVGCDLDFDLAGPTVHAAAVLGYEGWLAGYQMAFDTAKSKLAQNNFALGYKAGDFQLHTNVND-GTEFGGSIYQKVNGQLETAV
S.salar             ALDSSFVPNTGKKSAKLKTGYKRDFINLGCDLDFDMAGPTVHAAAVLGYEGWLAGYQMAFDTAKSKLAQNNFALGYKAGDFQLHTNVND-GTEFGGSIYQKVNCHLETAI
A.thaliana          IVQAKL---PDHKSGKAEVQYFHDYAGISTSVGFT-ATPIVNFSGVVGTNGLSLGTDVAYNTESGNFKHFNAGFNFTKDDLTASLILNDKGEKLNASYYQIVSP--STVV
O.sativa            ILSLVV---PDQRSGKLELQYLHEYAGINASVGLN-SNPMVNLSGVFGSKELSVGVDVAFDTATSNFTKYNAALSLTNSDLIASLHLNNHGDTLIASYYHLVKHHSNTAV
Clustal Consensus    :.  .     ::*.* :  : ::   :  .: .  : * :   .*.. :    * :::::* . .: : * .:  .  *:     :*:  *  : .* *: *.   .* :

                            230       240       250       260       270       280       290
                    ....|....|....|....|....|....|....|....|....|....|....|....|....|....|....|....
H.sapiens           NLA--WTAGSNNTRFGIAAKYMLDCRTSLSAKVNNASLIGLGYTQTLRPGVKLTLSALIDGKNFSAGGHKVGLGFELEA
C.griseus           NLA--WTAGSNNTRFGIAAKYKLDCRTSLSAKVNNASLIGLGYTQTLRAGVKLTLSALIDGKNFNAGGHKVGLGFELEA
B.taurus            NLA--WTAGSNNTRFGIAAKYKLDCRTSLSAKVNNASLIGLGYTQTLRPGVKLTLSALIDGKNFNAGGHKVGLGFELEA
S.scrofa            NLA--WTAGSNNTRFGIAAKYKLDCRTSLSAKVNNASLIGLGYTQTLRPGVKLTLSALIDGKNFSAGGHKVGLGFELEA
M.mulatta           NLA--WTAGSNNTRFGIAAKYKLDCRTSLSAKVNNASLIGLGYTQTLRPGVKLTLSALIDGKNFSAGGHKVGLGFELEA
O.cuniculus         NLA--WTAGSNNTRFGIAAKYKLDCRTSLSAKVNNASLIGLGYTQTLRPGVKLTLSALIDGKNFNAGGHKVGLGFELEA
M.musculus          NLA--WTAGSNNTRFGIAAKYKLDCRTSLSAKVNNASLIGLGYTQTLRPGVKLTLSALIDGKNFNAGGHKVGLGFELEA
R.norvegicus        NLA--WTAGSNNTRFGIAAKYRLDCRTSLSAKVNNASLIGLGYTQSLRPGVKLTLSALVDGKNFNAGGHKVGLGFELEA
B.mutus             NLA--WTAGSNNTRFGIAAKYKLDCRTSLSAKVNNASLIGLGYTQTLRPGVKLTLSALIDGKNFNAGGHKVGLGFELEA
M.brandtii          NLA--WTAGSNNTRFGIAAKYKLDCRTSLAAKVNNASLIGLGYTQTLRPGVKLTLSALIDGKNFNAGGHKVGLGFELEA
X.laevis            SLA--WSAGSNNTRFGIGAKYQLDSNTTISAKVNNASLIGVGYTQTLRPGVKLTLSALVNGKNFSAGGHKVGLGFELEA
C.mydas             NLA--WTAGSNNTRFGIATKYQLDENTSIAAKVNNASLIGIGYTQTLRPGVKLTLSGLVDGKNFSAGGHKVGLGFELEA
A.platyrhynchos*    NLA--WTAGSNNTRFGIAAKYQLDEKTSIVAKVNNASLIGIGYAHTLRPGIKLTLSGLIDGKNFSAGGHKVGLGFELEA
C.brachyrhynchos*   NLA--WTAGSNNTRFGIAGKYQLDEKTSIVAKVNNASLIGIGYTHALRPGVKLTLSGLIDGKNFSAGGHKIGLGFELEA
C.anna*             NLA--WTAGSNNTRFGIAAKYQMDEKTSVVAKVNNASLIGIGYTHALRPGVKLTLSGLIDGKNFSAGGHKVGLGFELEA
B.rhinoceros*       NLA--WTAGSNNTRFGLAAKYQLDEKTSIVTKVNNASLIGIGYTHALRPGVKLTFSGLIDGKNFSAGGHKVGLGFELEA
A.forsteri*         NLA--WTAGSNNTRFGIAAKYQLDEKTSIVAKVNNASLIGIGYAHALRPGVKLTLSGLIDGKNFSAGGHKVGLGFELEA
S.camelus*          NLA--WTAGSNNTRFGIAAKYQLDEKTSIVAKVNNASLIGIGYSHALRPGVKLTLSGLIDGKNFSAGGHKVGLGFELEA
M.gallopavo         NLA--WTAGSNNTRFGIAAKYQLDEKTSIVAKVNNASLIGIGYTLALRPGVKLTLSGLIDGKNFSAGGHKVGLGFELEA
G.gallus*           NLA--WTAGSNNTRFGIAAKYQLDEKTSIGAKVNNASLIGIGYTLALRPGVKLTLSGLIDGKNFSAGGHKVGLGFELEA
D.rerio             NLA--WTAGSNNTRFGIAAKYQLDKDSSVSAKVNNASLVGVGYTQSLRPGVKLTLSALIDAKNFNAGGHKVGMGFELEV
S.salar             NLA--WTAGSNNTRFGIGAKYQLDKNASLSAKVNNASLIGVGYTQALRPGVKLTLSALIDAKNFNAGGHKVGMGFELEA
A.thaliana          GAEISHNFTTKENAITVGTQHALDPLTTVKARVNNAGVANALIQHEWRPKSFFTVSGEVDSKAIDK-SAKVGIALALKP
O.sativa            GAELSHSFSRNESTLIFGSQHSLDPHTTVKARFNNYGMASALVQHEWRPKSLITISGEVDTKAIEK-STKVGLSLVLKH
Clustal Consensus         .   ::. : .. :: :*  ::: ::.** .:         *    :*.*. :: * :.  . *:*:.: *:
```

SUPPLEMENTAL FIGURE 3. Evolutionary conservation of cysteines in various VDAC-3 proteins. MSA of VDAC-3 sequence from various organisms generated using Clustal Omega (1,2). Cysteines are highlighted in yellow. The first ten organisms (higher mammals) show conservation of all six cysteines, while lower organisms and plants primarily have one conserved cysteine, which is replaced by a conserved serine in the first ten organisms. Organisms for which only partial sequence was available have been marked with *****.

SUPPLEMENTAL REFERENCES

1. Goujon, M., McWilliam, H., Li, W., Valentin, F., Squizzato, S., Paern, J., and Lopez, R. (2010) A new bioinformatics analysis tools framework at EMBL-EBI. *Nucleic Acids Res.* **38**, W695-699

2. Sievers, F., Wilm, A., Dineen, D., Gibson, T. J., Karplus, K., Li, W., Lopez, R., McWilliam, H., Remmert, M., Soding, J., Thompson, J. D., and Higgins, D. G. (2011) Fast, scalable generation of high-quality protein multiple sequence alignments using Clustal Omega. *Mol. Syst. Biol.* **7**, 539
